# Recent Developments in CaCO_3_ Nano-Drug Delivery Systems: Advancing Biomedicine in Tumor Diagnosis and Treatment

**DOI:** 10.3390/pharmaceutics16020275

**Published:** 2024-02-15

**Authors:** Chenteng Lin, Muhammad Akhtar, Yingjie Li, Min Ji, Rongqin Huang

**Affiliations:** 1 School of Pharmacy, Key Laboratory of Smart Drug Delivery (Ministry of Education), Huashan Hospital, Minhang Hospital, Fudan University, Shanghai 201203, China; 22111030050@m.fudan.edu.cn; 2 Department of Pharmaceutics, Faculty of Pharmacy, The Islamia University of Bahawalpur, Bahawalpur 63100, Pakistan; muhammad.akhtar@iub.edu.pk; 3 Shanghai Yangpu District Mental Health Center, Shanghai 200090, China; liyingjie0608@126.com

**Keywords:** calcium carbonate (CaCO_3_), synthesis methods, nano-drug delivery systems, tumor diagnosis, tumor treatment

## Abstract

Calcium carbonate (CaCO_3_), a natural common inorganic material with good biocompatibility, low toxicity, pH sensitivity, and low cost, has a widespread use in the pharmaceutical and chemical industries. In recent years, an increasing number of CaCO_3_-based nano-drug delivery systems have been developed. CaCO_3_ as a drug carrier and the utilization of CaCO_3_ as an efficient Ca^2+^ and CO_2_ donor have played a critical role in tumor diagnosis and treatment and have been explored in increasing depth and breadth. Starting from the CaCO_3_-based nano-drug delivery system, this paper systematically reviews the preparation of CaCO_3_ nanoparticles and the mechanisms of CaCO_3_-based therapeutic effects in the internal and external tumor environments and summarizes the latest advances in the application of CaCO_3_-based nano-drug delivery systems in tumor therapy. In view of the good biocompatibility and in vivo therapeutic mechanisms, they are expected to become an advancing biomedicine in the field of tumor diagnosis and treatment.

## 1. Introduction

Cancer, a serious health problem that threatens life and health security, has a perennial high incidence and mortality rate, resulting in nearly 10 million deaths worldwide in 2020 [1]. Many different therapeutic approaches have been proposed and tested for the treatment of cancer [2]. In the last few decades, nanoparticle-based drug delivery systems have gained great attention in chemotherapy due to their distinctive properties and potential applications in various cancers [3,4]. Nano-drug delivery systems consist of a combination of drug and medicinal materials with sizes in the range from 1 to 100 nm [5]. Nano-drug delivery systems can effectively improve the bioavailability, stability, and in vivo pharmacokinetic properties of drugs with high drug loading and can control the process of drug release [6,7]. The traditional theory of passive targeting is dominated by the enhanced permeability and retention (EPR) effect, which suggests that nano-drug delivery systems are able to enrich tumor tissues by virtue of their specific sizes. This is due to the specific physiological characteristics of the tumor region. The hyperpermeability of tumor neovascularization results in enhanced permeability of nanoparticles into the tumor mesenchyme, while impaired lymphatic drainage limits nanoparticle clearance, leading to enhanced retention [8,9]. As more and more novel targeting strategies have been proposed, researchers are now able to perform specific biologically or chemically targeted modifications on the surface of the nano-drug delivery system, which is referred to as the active targeting strategy, allowing the nanoparticles to be specifically targeted to the tumor site, enhancing therapeutic efficacy and reducing toxicities [10].

Based on the classification of different types of medicinal materials, we can classify the existing nanomedicine delivery systems into two categories: organic and inorganic, depending on whether they contain organic components or not [11]. Inorganic nanomaterials, including gold nanoparticles [12], silicon-based nanoparticles [13], iron-based nanoparticles [14], and calcium-based nanoparticles [15], have shown promising applications in tumor treatment and diagnosis due to their ease of preparation, high drug delivery rate, good biocompatibility, and easy surface modification compared to other nanomaterials.

**Figure 1:**
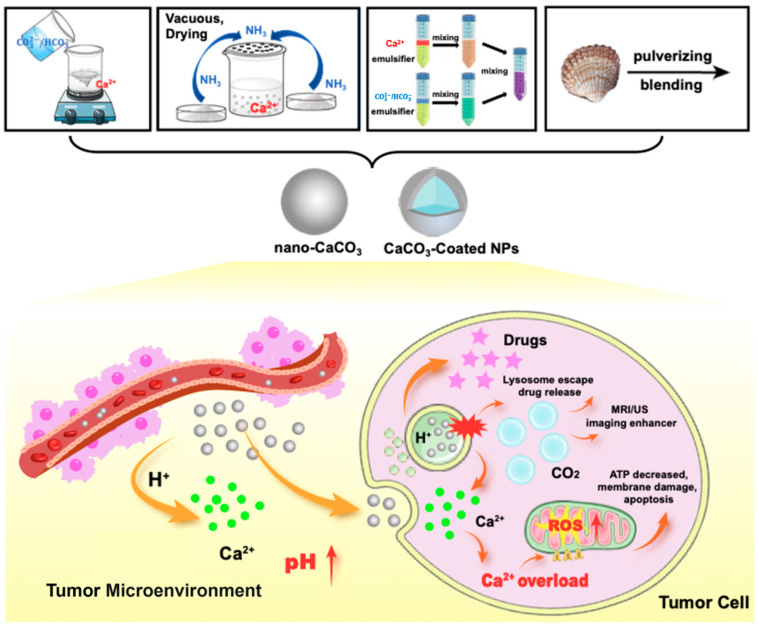
The synthetic methods, effects, and mechanisms of CaCO_3_ nanoparticles in cancer diagnosis and treatment.

Calcium carbonate (CaCO_3_) is a very common inorganic compound in nature and has wide applications in the pharmaceutical and chemical industries [16,17]. CaCO_3_ exists in nature mainly in crystalline and amorphous forms, and the crystals include aragonite, vaterite, and calcite, while the amorphous type is divided into the unstable amorphous calcium carbonate (ACC) phase and hydrated metastable forms [18,19,20]. In cancer treatment, CaCO_3_ nanoparticles have shown outstanding advantages due to their high biocompatibility, low toxicity, low-pH responsiveness, and low production cost [21]. Due to their good drug encapsulation rate, CaCO_3_ nanoparticles can effectively load small molecules and biomolecules for cancer therapy [22,23]. As shown in Figure 1, CaCO_3_ nanoparticles prepared by a series of different physical and chemical methods can decompose efficiently and releases the encapsulated drugs into the tumor tissues in a targeted manner, thus achieving targeted drug delivery and minimizing the leakage of drugs in the physiological environment of the organism [24,25,26]. Meanwhile, the surface mineralization strategy of CaCO_3_ nanoparticles has also shown great application value [27]. In addition, Ca^2+^ in CaCO_3_ and the CO_2_ generated from its decomposition have also shown important roles in the field of tumor therapy. The “calcium overload” effect [28] based on the significant increase in Ca^2+^ concentration in tumor cells can disrupt the normal mitochondrial function of tumor cells. CaCO_3_ is also critical for the regulation of acidity in the tumor microenvironment and CO_2_ release for drug release as well as imaging enhancement. Given the outstanding characteristics of CaCO_3_ nanoparticles in tumor therapy and the advantages of their nano-drug delivery system, we will explore and discuss the mechanisms and application progress of the CaCO_3_-based nano-drug delivery system in tumor therapy in more detail here.

In this review, we first summarize several major methods for synthesizing CaCO_3_ nanoparticles, such as chemical precipitation, gas diffusion, and microemulsion. Through these methods, CaCO_3_ nanoparticles with certain morphological characteristics and particle size distribution can be successfully synthesized, and different drug molecules or nanostructures can be added during the synthesis process, which can be efficiently encapsulated at the same time with the help of reactions, thus constructing CaCO_3_-based nano-drug delivery systems. Next, we analyzed the mechanisms of CaCO_3_ nanoparticles in tumor therapy and diagnosis in detail in combination with the existing constructed nano-drug delivery systems. Finally, we summarize and conclude the current CaCO_3_-based nano-drug delivery systems from the perspectives of encapsulated small molecules, biomolecules, and surface mineralization and provide an outlook on future development. We hope that this review will provide a more systematic overview of CaCO_3_-based nano-drug delivery systems and inspire researchers to design and develop novel related systems for safer and more efficient treatment of tumors.

## 2. The Synthesis Methods for CaCO_3_ Nanoparticles

For the preparation of CaCO_3_ nanoparticles, the main principle is to generate CaCO_3_ precipitates by mixing solutions containing Ca^2+^ (e.g., CaCl_2_ and Ca(NO_3_)_2_) with solutions containing CO_3_^2−^ (e.g., Na_2_CO_3_ and NaHCO_3_) [26]. By related particle size control methods, the prepared CaCO_3_ nanoparticles all showed a good nanoscale range. Based on this, the nano-drug delivery system based on CaCO_3_ can be obtained by further drug encapsulation and surface modification [29]. In addition, it is also possible to directly use bio-based materials containing CaCO_3_ such as cockle shells present in nature for the preparation of CaCO_3_ nanoparticles [30]. Given below is the description of the synthesis process of the CaCO_3_ nanoparticles in terms of different preparation methods.

### 2.1. Chemical Precipitation Method

Chemical precipitation is the direct reaction of Ca^2+^ and CO_3_^2−^ in solution under stirring conditions to produce CaCO_3_ nanoparticles [31]. Compared to other methods, chemical precipitation is simple and has a shorter reaction time. However, it should be noted that when CaCO_3_ nanoparticles of a specific size and shape need to be obtained, polyelectrolyte is usually added to interact with Ca^2+^ ions to control the nucleation and growth process of CaCO_3_. At the same time, for the reaction conditions, including the initial concentration of reactants, pH, and temperature, different choices will lead to the production of different CaCO_3_ crystals [32]. Therefore, although the chemical precipitation method is simpler than others, it has many influencing factors. In order to obtain the ideal nanoparticles, special attention should be paid to the changes in nucleation and crystal growth during the reaction.

Table 1 summarizes some of the representative literature on the preparation of CaCO_3_ nanoparticles using chemical precipitation methods, including information on raw materials, brief methods, and characterizations. By the direct chemical precipitation process, this method can achieve effective drug loading and surface mineralization modification for some drug molecules and even nanoparticles.

**Figure 2:**
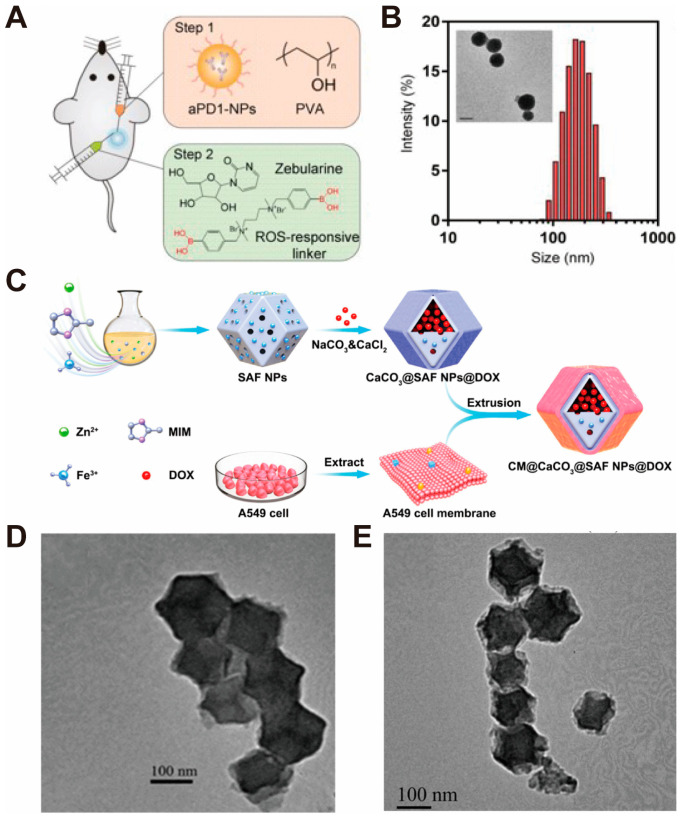
Example diagrams associated with the chemical precipitation method for the preparation of CaCO_3_ nanoparticles. (**A**) Schematic illustration of ROS/H^+^ dual-bioresponsive drug delivery depot. (**B**) Particle size distribution of aPD1-loaded CaCO_3_ NPs measured by dynamic light scattering (DLS) and morphology observed by transmission electron microscopy (TEM), where the scale bar is 100 nm. Adopted with permission from Ref. [34]. 2023, Chenteng Lin. (**C**) The above figure reveals the preparation of CM@CaCO_3_@SAF NPs@DOX nanoplatforms. (**D**) The TEM image of SAF NPs. (**E**) The TEM image of CaCO_3_@SAF NPs@DOX. Scale bars: 100 nm. Adopted with permission from Ref. [35]. 2023, Chenteng Lin.

Zhang and Hou et al. [33] used soluble starch as a growth platform for CaCO_3_ nanoparticles by adding a mixture of CaCl_2_ and soluble starch solution in a flask and stirring. Then, the Na_2_CO_3_ solution was quickly added to it, and the reaction was stirred vigorously. Finally, the resulting nanoparticles were collected by centrifugation. Ca^2+^ caused different changes in conformation of the added soluble starch. At this time, the starch with different conformation can in turn direct the arrangement of CaCO_3_ crystals after the addition of CO_3_^2−^. The X-ray diffraction pattern results showed that CaCO_3_ nanoparticles were a calcite/vaterite mixture, which was more soluble than other polymorphs and suitable for degradation in an acidic environment. Gu and Lu et al. [34] used PEG-P(Glu) copolymers to interact with Ca^2+^ and CO_3_^2−^ to prepare CaCO_3_ nanoparticles loaded with anti-PD1 antibodies (aPD1). The carboxyl group on glutamic acid (Glu) was able to interact with Ca^2+^ to prevent the generation of oversized CaCO_3_ nanoparticles, while the PEGylated structure was able to avoid the inter-agglomeration of nanoparticles. The average size of the nanoparticles was ~100 nm, and the aPD1 encapsulation efficiency was ~50% (Figure 2A,B).

Zhang et al. [35] successfully prepared SAF NPs@DOX nanoparticles covered with CaCO_3_ by surface mineralization modification of the synthesized CaCO_3_@SAF NPs@DOX by chemical precipitation (Figure 2C). The aqueous SAF NPs@DOX solution was mixed with a CaCl_2_ solution and stirred, and then the Na_2_CO_3_ solution was mixed to the above reaction system with continuous stirring. After that, the CaCO_3_-coated SAF NPs@DOX were finally obtained by separating the mixture by centrifugation. Transmission electron microscopy (TEM) imaging results showed that the CaCO_3_@SAF NPs@DOX display a homogeneous polygonal morphology with a diameter of ~120 nm. The CaCO_3_-mineralized shell can be detected from the TEM images clearly, and the layer of mineralization has a thickness of about 15 nm (Figure 2D,E).

### 2.2. Gas Diffusion Method

The preparation of CaCO_3_ nanoparticles by a gas diffusion reaction [24] is based on the volatilization and diffusion of CO_2_ in a vacuum-closed system and dissolution in the solution containing Ca^2+^. The precursors used in this method are mainly ethanolic solutions of CaCl_2_ and (NH_4_)_2_CO_3_ or NH_4_HCO_3_ solid particles; the latter decompose under vacuum to generate CO_2_. The gas diffusion method is also relatively simple to operate. The thermodynamically unstable amorphous calcium carbonate (ACC) phase can be prepared efficiently by the gas diffusion method and by controlling the water content in the reactive organic solvent [48]. In contrast to the traditional crystalline form of CaCO_3_, ACC can be easily obtained on a nanoscale. ACC nanoparticles have active acid responsiveness and thermal instability and are easily hydrolyzed in the intracellular environment, which has profound implications for the preparation of a preloaded drug delivery vector and therapeutic system for biomedical applications [48,49].

Table 2 summarizes some of the representative literature on the preparation of CaCO_3_ nanoparticles using the gas diffusion method. By adding different drug molecules or nanoparticles to the CaCl_2_ solution and mixing them well, it is possible to generate CaCO_3_ nanoparticles while achieving efficient loading of the drug. Moreover, by adjusting the temperature and time, CaCO_3_ nanoparticles generated by this method have a desirable particle size distribution and excellent morphological characteristics.

Han et al. [74] dissolved CaCl_2_ in a beaker containing anhydrous ethanol and covered the beaker with a thin film with holes on its surface. They added NH_4_HCO_3_ into another beaker and dried them in a vacuum environment for 24 h to prepare spherical CaCO_3_ nanoparticles with diameters of about 100 nm. On this basis, by adding different compound molecules, such as doxorubicin (DOX), curcumin (Cur), tizaramine (TPZ), indole green (ICG), and glucose oxidase (GOD) to the CaCl_2_ anhydrous ethanol solution, the researchers achieved efficient loading of these molecules by CaCO_3_ nanoparticles with the help of gas diffusion–reaction, using CO_2_ decomposed from ammonium bicarbonate to bind with Ca^2+^ in the solution.

**Figure 3:**
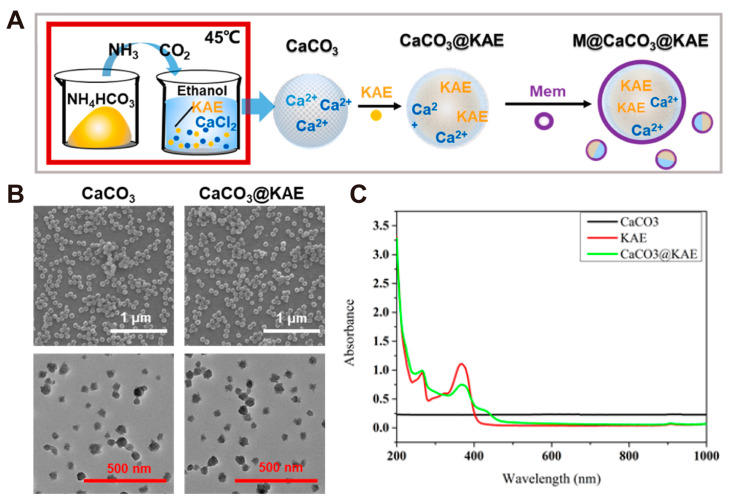
Schematic illustration and characterizations of KAE-loaded M@CaCO_3_@KAE NPs. (**A**) Schematic illustration of the synthesis process of M@CaCO_3_@KAE NPs. (**B**) TEM images of the CaCO_3_ NPs and CaCO_3_@KAE NPs. (**C**) UV–vis absorbance spectra of CaCO_3_ NPs, KAE, and CaCO_3_@KAE NPs. Adopted with permission from Ref. [61]. 2023, Chenteng Lin.

Chu and Zheng et al. [61] added kaempferol-3-O-Rutinoside (KAE), a biosafety flavonoid that can effectively promote Ca^2+^ influx and disrupt calcium homeostasis regulation of tumor cells, into a CaCl_2_ anhydrous ethanol solution. CaCO_3_@KAE nanoparticles were prepared after being placed together with NH_4_HCO_3_ in a vacuum-drying environment for 4 days (Figure 3A). The diameter of CaCO_3_@KAE nanoparticles is ~100 nm and has a uniform morphology with good dispersion (Figure 3B). Calculating the drug-loading amount of KAE in the nanoparticles through UV–vis absorbance spectra, the drug content in the nanoparticles is 18.57 ± 1.34%, which indicates that CaCO_3_ nanoparticles can achieve the effective load of KAE (Figure 3C).

### 2.3. Microemulsion Method

The precipitation in nano-droplets of microemulsions is one of the common methods for the synthesis of CaCO_3_ nanoparticles. Researchers have studied the preparation of CaCO_3_ nanoparticles by microemulsion methods such as reversed-phase microemulsion and biphasic microemulsion. Typically, in water-in-oil (W/O) systems, the precipitation of CaCO_3_ begins with the mixing of two micellar solutions containing Ca^2+^ and CO_3_^2−^. In this microreactor, the nucleation and growth of CaCO_3_ are limited, and the agglomeration of CaCO_3_ particles is effectively inhibited. Therefore, CaCO_3_ nanocrystals with particularly narrow size distribution, polycrystalline content, and morphology can be synthesized [75,76]. At the same time, the supported drug is dissolved in the corresponding water phase/oil phase, which can achieve the efficient loading of the drug while synthesizing CaCO_3_ nanoparticles. Compared with the previous methods, the operation of the microemulsion method is more complex, but its requirements for reaction conditions are not as strict. And there are more possibilities for the load selection of water-soluble/water-insoluble drugs when constructing a nano-drug delivery system.

Table 3 summarizes some of the representative literature on the preparation of CaCO_3_ nanoparticles by the microemulsion method, which lists the raw materials, the specific microemulsion method, and the size of the synthesized nanoparticles.

Khan et al. [80] prepared CaCO_3_ nanoparticles encapsulated with cisplatin by the water-in-oil (W/O) microemulsion method. First, they prepared two types of water-in-oil microemulsions. In brief, the CaCl_2_ solution was dispersed in the oil phase to produce an oil-in-water calcium inverse microemulsion. A carbonate phase emulsion was prepared by a dispersive Na_2_CO_3_ aqueous solution in an oil phase. A prodrug solution of cisplatin dissolved in chloroform and 1,2-dioleoyl-sn-glycero-3-phosphate (sodium salt) (DOPA) were simultaneously added to the carbonate phase. After 20 min of mixing alone, the two phases were mixed and after 30 min, anhydrous ethanol was mixed to disrupt the microemulsion system. The nanoparticles were then centrifuged, washed with anhydrous ethanol to remove surfactant and cyclohexane, and collected. The average diameter of the nanoparticles as measured by dynamic light scattering (DLS) was 217 ± 20 nm; the zeta potential was −23.7 ± 2 mV, and the average polydispersity index was 0.187. The transmission electron microscope (TEM) results indicated that the nanoparticles (NPs) were spherical, with a core of CaCO_3_ nanoparticles.

In addition to the W/O microemulsion method, other methods generally based on emulsion techniques including the water-in-oil-in-water (W1/O/W2) double emulsion [86] and oil-in-water (O/W) microemulsion method by using a High-Pressure Homogenization (HPH) [29] have also been used for the preparation of CaCO_3_ nanoparticles.

**Figure 4:**
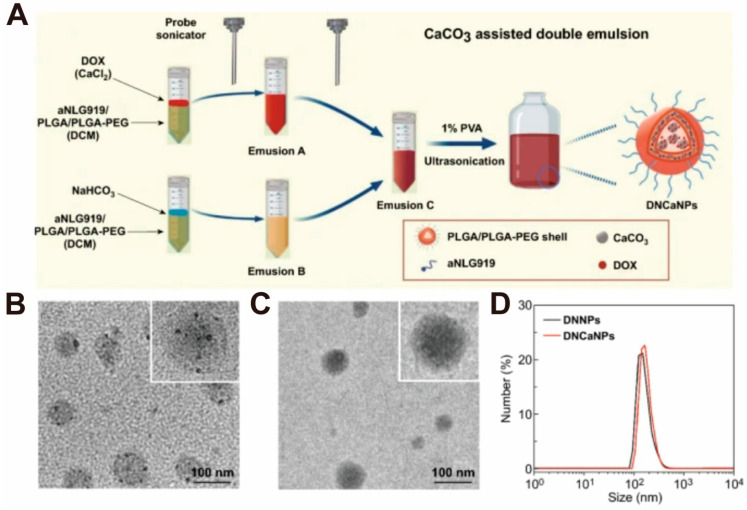
Example diagrams associated with the microemulsion method for the preparation of CaCO_3_ nanoparticles. (**A**) Schematic illustration of the preparation process of DNCaNPs via double emulsion method. (**B**) TEM images of DNCaNPs. (**C**) TEM images of DNNPs. (**D**) DLS size distribution of DNNPs and DNCaNPs. Adopted with permission from Ref. [83]. 2023, Chenteng Lin.

Liu and Feng et al. [83] adopted an improved W1/O/W2 double emulsification method to prepare CaCO_3_ nanoparticles (Figure 4A). Briefly, to prepare emulsions A (CaCl_2_ phase) and B (NaHCO_3_ phase), first, a CaCl_2_ aqueous solution (containing DOX) and a NaHCO_3_ aqueous solution were added to the dichloromethane solution containing alkylated NLG919 (Anlg919), poly(lactic-co-glycolic acid) (PLGA), and poly(lactic-co-glycolic acid)-polyethylene glycol (PLGA-PEG) followed by 99 pulses using the probe sonicator for sonication. Then, emulsions A and B were mixed and 99 pulses were performed using the probe sonicator to obtain emulsion C. Emulsion C was added dropwise to the polyvinyl alcohol (PVA) solution under sonication in a water bath and left overnight to remove the dichloromethane by evaporation. Afterward, the obtained DNCaNPs were washed by centrifugation to remove excess PVA, free drug, and large particles. With the help of TEM, it was observed that there were obvious dark spots of CaCO_3_ nanoparticles in the DNCaNPs, and the content of CaCO_3_ was 17.6% as measured by a commercial calcium colorimetric assay kit (Figure 4B–D).

### 2.4. Bio-Based Preparation Method

Bio-based materials with CaCO_3_ as the main component are widely distributed in nature, so the preparation of CaCO_3_ nanoparticles from biobased CaCO_3_ materials is a cheap and environmentally friendly method. Through multi-step physical grinding, the original biobased material can be sorted into powders at different micron/nanometer levels for further applications. The production cost of this method is the lowest of all methods and can allow industrial scale-up production.

Hamidu et al. [87] have successfully prepared 30 nm CaCO_3_ nanoparticles using commercially available shells (*Anadara granosa*) and showed uniform spherical shape. They first washed the shells with water and then boiled and dried them. The dried shells were further cleaned in water with a squeezed banana pelt agent, removing the impurities from the shells. Subsequently, the shells were crushed into powder by a rotary crushing mixer and sieved with a pore size of 75 μm. Then, the powder was mixed to deionized water with dodecyl dimethyl betaine (BS-12) and stirred. After cooling, the solution was filtered and precipitated and dried. The high-resolution transmission electron microscopy (HRTEM) results showed that the CaCO_3_ nanoparticles display a homogeneous spherical morphology, with a size of 35.5 nm.

## 3. The Effects and Mechanisms of CaCO_3_ Nanoparticles in Cancer Diagnosis and Treatment

CaCO_3_ nanoparticles have significant advantages such as pH sensitivity and a high Ca^2+^ content. When the nanomaterials containing CaCO_3_ enter the low-pH environment of the tumor tissue, CaCO_3_ decomposes, neutralizes acidic H^+^, generates large amounts of Ca^2+^, and generates CO_2_. When these substances enter tumor cells, they can specifically regulate the metabolic process of tumor cells by their special properties. Below, we will discuss some of the effects and mechanisms of CaCO_3_ nanoparticles in cancer diagnosis and treatment from different aspects.

### 3.1. Acidity Modulation

The tumor tissue microenvironment has a lower pH compared to normal physiological tissues, which allows acid-sensitive nanomaterials to specifically respond to the high proton concentrations within the tumor tissue, thus enabling the neutralization of H^+^ in the tumor microenvironment (TME) and the pH-responsive release of the loaded drugs. CaCO_3_, a classical high-pH sensitivity compound, plays an essential factor in reprogramming the acidic environment of the TME, and that is why CaCO_3_-based nanoparticles have a modulating effect on the immune environment of tumors [88,89]. Their acidic degradation products include only Ca^2+^ and CO_2_ [27,43]. While significantly enhancing the efficacy of other oncology therapies, it is unlikely to have adverse effects [57,62,90].

Liu and Feng et al. [91] constructed AIM NPs (acidity-IDO1-modulation nanoparticles, 4PI-Zn@CaCO_3_) based on CaCO_3_ nanoparticles. To investigate the modulatory effect of AIM NPs on the TME, the investigators used commercial pH microelectrodes to directly measure the pH in mouse CT26 tumors and assess the neutralizing efficacy of CaCO_3_, 4PI-Zn, and 4PI-Zn@CaCO_3_ on the acidity in the TME. The results indicated that the pH of these tumors increased significantly 24 h after intravenous injection of AIM and CaCO_3_ nanoparticles, from pH 6.6 at about 0 h to pH 7.0 at 24 h, compared with that before the corresponding pre-injection period. In contrast, the intravenous injection of 4PI-Zn nanoparticles had a minimal effect on tumor pH, and even the pH at 0 h dropped from 6.6 to 6.5. The results suggested that both AIM and blank CaCO_3_ nanoparticles are effective tumor acid regulators.

Gu et al. [92] prepared an aCD47@CaCO_3_ nanoparticles-based bioresponsive fibrin gel for in situ spraying at postoperative tumor resection sites, and after spraying the gel, was able to remove H^+^ from surgical wounds and polarize tumor-associated macrophages (TAMs) to an M1-like phenotype, while inducing macrophage phagocytosis of tumor cells through the blockade of the interaction between CD47 and SIRPα to enhance anti-tumor T cell responses. The researchers investigated the effect of CaCO_3_ as a proton scavenger in aCD47@CaCO_3_ nanoparticles on the pH change in phosphate buffer solution (PBS) in vitro. After the addition of aCD47@CaCO_3_ nanoparticles to PBS solutions with original pH values of 6.0 and 6.5, the pH of the solution progressively increased to about 7.4 as time progressed from 0 h to 24 h.

### 3.2. Calcium Overload

Calcium overload, as the name implies, is the accumulation of free Ca^2+^ in the cell cytoplasm resulting in an abnormally high concentration of Ca^2+^, which can lead to severe cellular damage or even cell death [93]. Normally, calcium signaling acts as a second messenger of cellular signaling to control important physiological processes by modulating the activity of specific targets, which are essentially achieved in response to fluctuations in local calcium concentrations [94]. However, any changes in intracellular Ca^2+^ concentrations induced by external factors can disrupt normal calcium signaling and thus affect various cellular activities. For example, under oxidative stress, the cellular regulation of Ca^2+^ is reduced, as evidenced by abnormal calcium channel functions such as calcium pumps, and intracellular calcium ion concentrations are difficult to reregulate to a normal state, leading to a calcium overload [95]. Once intracellular calcium overload occurs, it will trigger the disruption of Ca^2+^ homeostasis in the mitochondria, resulting in decreased mitochondrial membrane potential and adenosine triphosphate (ATP) levels, osmotic swelling, and mitochondrial respiratory disorder and eventually lead to mitochondria-related cell damage and apoptosis [96].

As an ideal Ca^2+^ donor, CaCO_3_-based intracellular calcium nanoreactors have good low-pH responsiveness in the tumor microenvironment and have been exploited for calcium overload-mediated therapy. Ding et al. [65] prepared a multichannel Ca^2+^ nanomodulator (CaNM_CUR+CDDP_), loaded with cisplatin (CDDP) and curcumin (CUR) to enhance calcium overload-induced therapy. After administration, PEGylated CaNM_CUR+CDDP_ (^PEG^CaNM_CUR+CDDP_) aggregates in the tumor and enters into tumor cells, inducing multi-level mitochondrial disruption under the synergistic effects of massive Ca^2+^ burst release, CUR inhibition of calcium efflux, and chemotherapy of CDDP, thereby significantly enhancing mitochondria-targeted suppression of the tumor.

**Figure 5:**
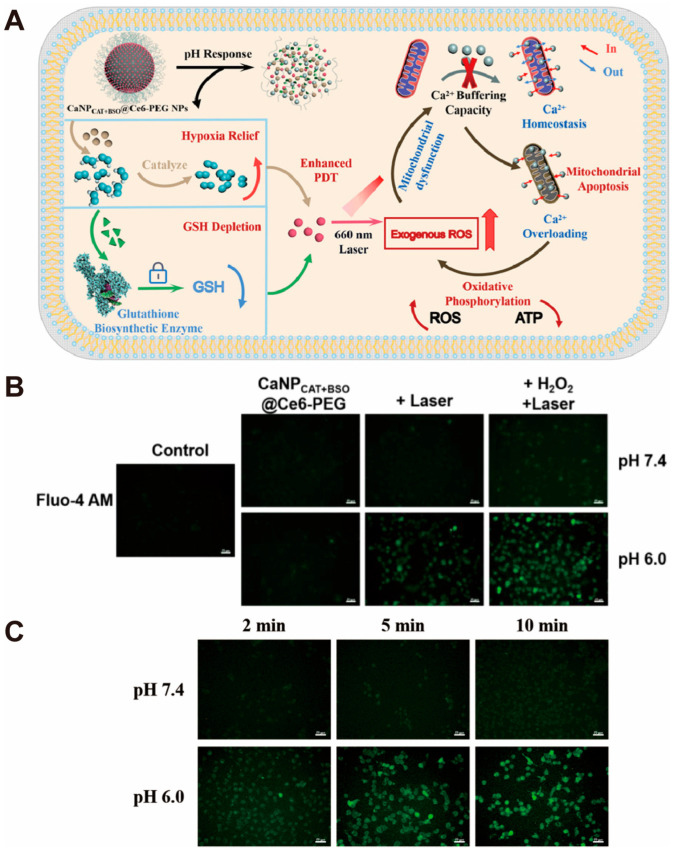
Example of nanosystems constructed from CaCO_3_ nanoparticles for calcium overload of tumor cells. (**A**) Schematic illustration of the CaNP_CAT-BSO_@Ce6-PEG NPs for PDT-enhancing and mitochondrial Ca^2+^ overload synergistic therapy. (**B**) The CLSM images demonstrate the difference in intracellular Ca^2+^ content after different groups of drug treatments. (**C**) The CLSM images demonstrate the detection of intracellular Ca^2+^ content after irradiation for different times. Adopted with permission from Ref. [97]. 2023, Chenteng Lin.

Dong and Song et al. [97] designed a CaCO_3_-based nanoplatform (CaNP_CAT+BSO_@Ce6-PEG) for the synergistic treatment of enhanced photodynamic therapy (PDT) and calcium overload (Figure 5A). In acidic TME, CaCO_3_ decomposes and releases loaded drugs, including catalase (CAT), buthionine sulfoximine (BSO), the photosensitizer Chlorin e6 (Ce6), and Ca^2+^. CAT and BSO significantly reverse the hypoxic microenvironment of tumors, significantly enhancing the PDT effect. The ^1^O_2_ produced by PDT kills tumor cells directly and disrupts the mitochondrial calcium homeostasis, leading to a mitochondrial calcium overload. The increased concentration of Ca^2+^ inhibits the production of ATP in mitochondria, triggering mitochondrial dysfunction and thus accelerating cell death (Figure 5B,C). With the combined effect of enhanced PDT and calcium overload, the nanoparticles exhibited a significant synergistic tumor-suppressive effect.

### 3.3. Facilitating Lysosome Escape and Intracellular Drug Release

When a nano-drug delivery system is uptaken by a cell, how to achieve efficient lysosomal/endosomal escape is a critical step for its function [98]. Currently, the proton sponge theory has a place in the research of the mechanism of lysosomal/endosomal escape [99]. Researchers suggest that when substances with high buffering capacity and adaptability enter the endosome, in order to buffer the acidic environment of the endosome, a large number of protons will be taken up, resulting in a difference in transmembrane voltage and a large amount of Cl- and H_2_O in the cytoplasm influx, which ultimately leads to the lysosomal swelling and rupture [100]. This is referred to as the proton sponge effect. CaCO_3_ nanoparticles are widely regarded as a class of proton nanosponge materials due to their efficient acid response properties [101].

Peng et al. [79] prepared a CaCO_3_ liposome nanoparticle (LCC) loaded with curcumin (CUR), which has a high sensitivity to the lysosomal low-pH environment. Because of the pH sensitivity of CaCO_3_, the LCC swelled in the lysosomal acidic environment and rapidly released the encapsulated CUR. Further studies showed that the accumulation of CUR in the cytoplasm of LCC was due to the liposomes’ pH sensitivity, leading to effective lysosomal escape and the release of CUR. The breakdown of CaCO_3_ resulted in the presence of higher concentrations of Ca^2+^ in the lysosomes and an increase in osmolality and allowed the influx of water from the cytoplasm into the lysosome. Eventually, CUR, which is rapidly released in an acidic environment, is transported into the cytoplasm after lysosome rupture. Thus, LCC can effectively promoted the accumulation of CUR in the cytoplasm.

**Figure 6:**
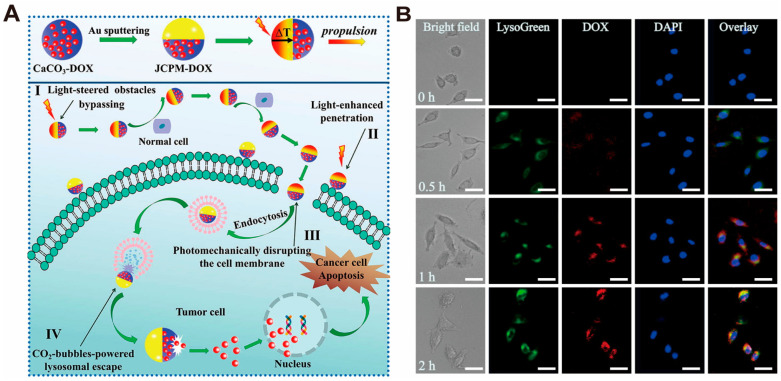
Examples of nanosystems constructed from CaCO_3_ nanoparticles for facilitating lysosome escape and intracellular drug release. (**A**) Schematic illustration of the mechanism of the light/gas cascade-propelled JCPMs. (**B**) The CLSM images of JCPMs-DOX lysosome escape. Green: lysosomes, blue: nuclei, red: DOX loaded in JCPMs. Scale bar: 20 μm. Adopted with permission from Ref. [102]. 2023, Chenteng Lin.

In the low-pH environment, CaCO_3_ decomposition is not only able to produce Ca^2+^ but also to generate CO_2_ gas for gas-driven reactions in tumor cells. When the nano-drug delivery system based on CaCO_3_ enters the lysosome, CaCO_3_ decomposes into large volumes of CO_2_ in the low-pH environment, which drives the platforms to penetrate the lysosome barrier and release the drugs. Moreover, because of the driving effect of gas, the drug release can be accelerated [102]. Jiao and Zhang et al. [102] designed a cascaded Near-Infrared (NIR) light/gas-driven Janus CaCO_3_ particle micromotor (JCPM) to overcome the biological barrier at various stages after the system enters the body to achieve active drug delivery targeting tumor cells (Figure 6A). When the JCPM enters the lysosome, large amounts of CO_2_ are rapidly produced during CaCO_3_ decomposition in an acidic lysosomal microenvironment, and the pressure in the lysosome increases dramatically, promoting gas-driven lysosomal escape and DOX release induced by CaCO_3_ decomposition (Figure 6B).

### 3.4. Tumor Immunomodulation

Previous studies have shown that calcium-based materials can enhance anticancer immunity [103,104]. Ca^2+^ has significant advantages in tumor immunotherapy such as (i) inducing immunogenic cell death (ICD) in tumor cells [64]; (ii) increasing autophagic efficiency [41]; and (iii) promoting the polarization of M2 macrophages to M1 macrophages [105]. Many CaCO_3_-based nanosystems have been constructed to activate immunotherapy against cancer.

Tumor immunogenic cell death (ICD) activates damage-associated molecular patterns (DAMPs) that promote the maturation of dendritic cells (DCs) and proliferation of cytotoxic T lymphocytes, thereby activating anti-tumor immune responses [106]. Recently, Ca^2+^ has been shown as a novel ICD-inducing factor [107], and imbalances of mitochondrial calcium homeostasis can modulate reactive oxygen species (ROS) production, which stimulates damage-associated molecular patterns (DAMPs) to cause ICD and finally activate defensive anti-tumor immunity. Zheng et al. [64] synthesized a multifunctional Ca^2+^ nanoregulator, ^PEG^CaCUR, which is a pH-sensitive PEG-modified CaCO_3_ nanoparticle loaded with curcumin (CUR). ^PEG^CaCUR is capable of releasing Ca^2+^ and CUR in a low-pH intracellular environment. CUR promotes Ca^2+^ release from the endoplasmic reticulum to the cytoplasm and inhibits Ca^2+^ efflux, inducing calcium overload, which in turn leads to apoptosis. After combining with the effect of ultrasound (US), it not only enhanced cellular uptake, but also promoted the influx of extracellular Ca^2+^, leading to an enhanced calcium overload and the upregulation of ROS levels.

Autophagy promotes phagocytosis and the presentation of antigen by dendritic cells during antigen processing [108,109]. However, the autophagic capacity of dendritic cells is frequently inhibited in TME [110], causing a severe reduction in the intensity of antigen presentation. It has been shown that the homeostasis of different intracellular ions controls the activity of a wide variety of enzymes/proteins, such as Ca^2+^, which play a key role in antigen presentation [111]. Thus, modulating calcium levels in dendritic cells has the promise of improving autophagy and thus enhancing immunotherapy. Ma and Wei et al. [46] developed a bionic approach to prepare graded ovalbumin@CaCO_3_ nanoparticles (OVA@NP) in the presence of a template of ovalbumin antigen. OVA@NP was able to efficiently transport antigen into dendritic cells and primitive lysosomes. Once OVA@NP entered the lysosome, the environment caused the rapid disintegration of CaCO_3_ material and was accompanied by bursts of CO_2_ production and a dramatic increase in pressure within the lysosome, leading to lysosomal disintegration. Moreover, the researchers also observed the formation of isolated/extended bowl-shaped phagosomes and typical double-membrane autophagosomes near the disintegrated lysosomes in NP-treated DCs. The bowl-shaped phagosomes wrapped around the cytoplasmic components to form an intact autophagic vesicle. Based on this phenomenon, the researchers presented the first evidence that physical stress generated by carbon dioxide induces autophagy via the Lc3/Beclin 1 pathway. In this process, residual OVA in lysosomes, together with cytoplasmic proteasomes, were wrapped by autophagosomes, which together promoted antigen cross-presentation in DC cells, induced the proliferation of CD8+ T cell, triggered a vigorous specific cytotoxic T lymphocyte (CTL) response, and ultimately led to significant tumor therapeutic effects.

Macrophages are involved in a multitude of biological processes, encompassing stimulation of infection, pathological progression, and maintenance of homeostasis [112,113], and are expressed in two main phenotypes, inflammation-promoting M1-type macrophages and anti-inflammation-promoting M2-type macrophages. Significantly, TAMs, the most abundant tumor-expanding immune cells, are often expressed as M2-type and promote immune escape and tumor metastasis [114,115]. So, regulating the polarization of macrophage phenotypes from M2 to M1 is essential for immunotherapy. The function of Ca^2+^ in regulating the macrophage polarization from M2 to M1 has been well reported [116,117], not the least of which is the construction of nano-drug delivery systems based on CaCO_3_. For example, Huo and colleagues [36] synthesized a CaCO_3_-DC biomineralized hydrogel vaccine by immobilizing membrane proteins of 4T1 cell-DC fusion cells (FPs) in a hydrogel. The addition of CaCO_3_ increased the pH of TME and facilitated the polarization from M2 to M1, which in turn reversed the immune-suppressive microenvironment and mitigated the immune-suppressive effects on T cells. In the experiments, the researchers observed an upregulation of CD80 (M1-type marker) expression and a downregulation of CD206 (M2-type marker) expression after IL-4+CaCO_3_ treatment. Similarly, Yang et al. [40] synthesized CaCO_3_-encapsulated Au nanoparticles (Au@CaCO_3_ NPs) as a stimulus for macrophage regulation. In contrast to AuNPs, which polarized macrophages to the M2-type, this study showed that coincubation of Au@CaCO_3_ NPs with macrophages resulted in cell rounding and induced secretion of the M1-type biomarker nitric oxide (NO), as well as TNF-α and IL-1β. A change in the macrophage polarization phenotype from M2 to M1 was observed after the coincubation of Au@CaCO_3_ NPs with M2-type macrophages formed by IL-10 induction. In summary, CaCO_3_ nanoparticles play a key role in polarizing to the M1-type macrophages, reversing the suppressive immune-microenvironment and facilitating tumor killing.

### 3.5. Magnetic Resonance Imaging and Ultrasound Contrast Enhancers

CaCO_3_ has been extensively studied as a pH-responsive material not only because of its suitability for targeted delivery of active drugs but also because of its ability to generate CO_2_ in the low-pH environment of typical tumor microenvironments, which provides a potential ultrasound contrast agent for detecting and imaging tumors [27,118].

**Figure 7:**
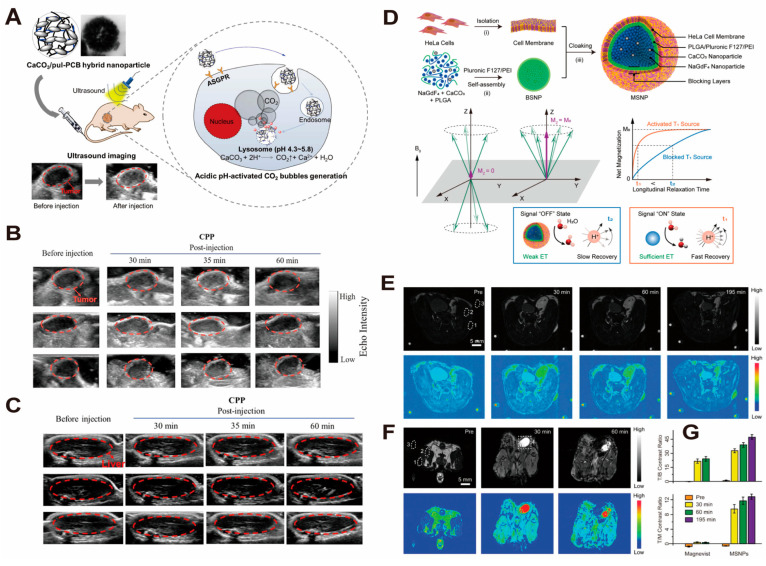
Examples of nanosystems constructed from CaCO_3_ nanoparticles for magnetic resonance imaging and ultrasound contrast enhancers. (**A**) Schematic illustration demonstrates the principle of CaCO_3_/pul-PCB (CPP) hybrid nanoparticles in contrast-enhanced US diagnosis of hepatocellular carcinoma. (**B**,**C**) show the difference in US imaging of tumor and liver before and after intravenous injection of CPP in vivo. Adopted with permission from Ref. [119]. 2023, Chenteng Lin. (**D**) Schematic illustration of the synthesis of MSNPs as smart contrast agents for T1-weighted MRI. (**E**–**G**) show T1-weighted MRI and corresponding pseudocolor images of tumor-bearing mice after intravenous injection of the same dose of MSNPs (**E**) and Magnevist (**F**). Tumor-to-background (T/B, (**G**), above) and tumor-to-muscle (T/M, (**G**), below) contrast based on the corresponding MRI images. The dotted circles represent the regions of interest: (1) tumor, (2) muscle, (3) background, and (4) bladder. Scale bars are 5 mm for all images. Adopted with permission from Ref. [121]. 2023, Chenteng Lin.

Luo and Zhang et al. [119] constructed CaCO_3_/pul-PCB (CPP) hybrid nanoparticles by using pul-PCB copolymer as a surface modifier, and the surface of the nanoparticles was modified by mineralization of Pullulan polysaccharides (Figure 7A). After injection, the nanoparticles can achieve efficient accumulation in the tumor tissue and decompose under acidic conditions, producing CO_2_ bubbles with echogenic effects that enhance ultrasonic signals. It was demonstrated that the contrast of the ultrasonic signal was strengthened 6 times at the tumor site within 35 min in tumor-bearing mice. In contrast, there is almost no change in signal in normal mice (Figure 7B,C). So, CPP nanoparticles were considered a prospective contrast material for the imaging of liver cancer.

Guo and Zhang et al. [120] developed a CaCO_3_-based diagnostic nanosystem for ultrasound and fluorescence dual-mode imaging. Under acidic conditions, the nanoparticles were able to generate CO_2_ to strengthen ultrasound imaging, and a large number of CO_2_ bubbles were generated in the tumor tissue after injecting CaCO_3_-DOX NPs intravenously into tumor-bearing mice under the effect of echo reflection from the ultrasound field. To investigate the possibility of CaCO_3_-DOX nanoparticles for in vivo ultrasound imaging of tumors, the researchers injected the nanoparticles by tail vein injection and subsequently performed ultrasound imaging. The results showed that the enhancement of ultrasound images at the tumor site was clearly observed after CaCO_3_-DOX nanoparticles injection, and this signal contrast enhancement lasted for nearly 100 min. Compared with conventional contrast agents (sulfur hexafluoride microbubbles for injection), CaCO_3_ nanoparticles can enter the tumor through the EPR effect and release CO_2_ in the low-pH microenvironment of the tumor for a longer duration, thus obtaining more accurate diagnosis results.

In addition to applications in ultrasonography, CaCO_3_-based nanosystems are also of use in magnetic resonance imaging (MRI) contrast. Liu et al. [121] reported a new type of contrast agent consisting of NaGdF_4_ and CaCO_3_ nanoparticles (Figure 7D). In the study, a “turn down” in MRI signal resulted from spatial confinement of the T1 source (Gd^3+^ ions) due to insufficient interaction between the plasma and the lattice. However, upon entering a low-pH environment, CaCO_3_ nanoparticles can produce CO_2_, which breaks the binding between NaGdF_4_ and CaCO_3_, allowing the MRI signal to be “turned on”. As shown in the figure, NaGdF_4_-CaCO_3_ nanoconjugates showed a significant enhancement of the contrast between the tumor site and the background tissue before and after intravenous injection, with the tumor-to-background (T/B) ratio reaching 48. Compared with the commercially available contrast agent, Magnevist, it provided a significant enhancement of the imaging contrast for the tumor site. (Figure 7E–G). This research represents remarkable progress in the creation of intelligent MRI nanoprobes, with key implications for the imaging of deep tissues and the diagnosis of specific cancers.

## 4. CaCO_3_-Based Nano-Drug Delivery Systems for Tumor Diagnosis and Treatment

Currently, CaCO_3_-based nano-drug delivery therapeutic systems have formed a relatively mature system and have shown their unique and extensive use in the delivery of various small-molecule drugs and biomolecules. In addition, CaCO_3_ can be covered on the surface of other nanostructures such as carbon quantum dots (CQDs) and metal–organic frameworks (MOFs) by surface mineralization to achieve CaCO_3_-based nano-medical delivery. In the following, we will introduce the construction of some recent CaCO_3_-based nano-drug delivery systems and their important roles in oncology therapy from three dimensions: small-molecule drugs, biomolecules, and surface mineralization. The preparation methods, physicochemical properties, and main findings of a part of CaCO_3_-based nano-drug delivery systems loaded with small-molecule drugs, biomolecule drugs, and surface mineralization are shown in Table 4.

### 4.1. Small-Molecule Drug Nano-Drug Delivery Systems

As an intrinsically biocompatible and pH-sensitive compound, various nano-CaCO_3_-based biomaterials have been widely explored for the construction of small-molecule drug delivery vehicles. A series of small-molecular drugs, including Doxorubicin (DOX) [82,132,133,134], Bleomycin (BLM) [62], Curcumin (Cur) [135], and methotrexate (MTX) [136], as well as small-molecule photosensitizers such as indocyanine green (ICG) [60] and Chlorin e6 (Ce6) [124], have been widely loaded into nano-delivery carriers constructed from CaCO_3_ for a variety of different oncology therapeutic approaches.

**Figure 8:**
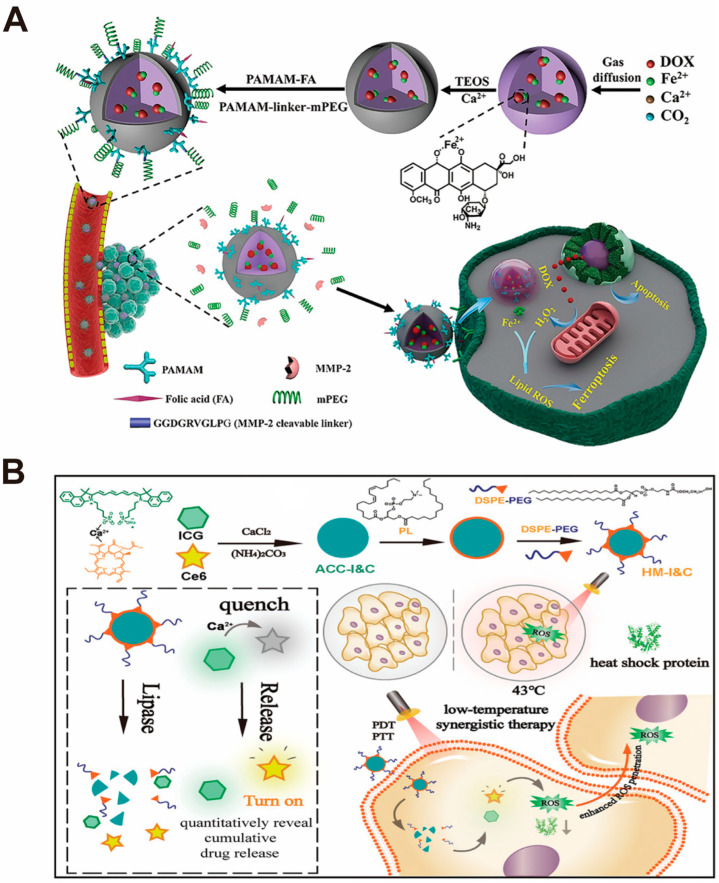
Examples of small-molecule drug nano-drug delivery systems constructed based on CaCO_3_ nanoparticles. (**A**) Schematic illustration of the synthesis and mechanism of ACC@DOX.Fe^2+^-CaSi-PAMAM-FA/Mpeg and its complementary ferroptosis/apoptosis-based therapeutic action. Adopted with permission from Ref. [90]. 2023, Chenteng Lin. (**B**) Schematic illustration of the synthesis and mechanism of HM-I&C for dual-channel imaging and low-temperature synergistic therapy. Adopted with permission from Ref. [54]. 2023, Chenteng Lin.

Yu and Luo et al. [90] designed a tumor-targeting amorphous calcium carbonate (ACC)-based nanoparticle for tumor-targeted iron death therapy (Figure 8A). DOX was first chelated with Fe^2+^ and then ACC encapsulated with Fe^2+^-DOX molecules was synthesized in one step by the gas diffusion method. Subsequently, an additional layer of SiO_2_-CaCO_3_ (CaSi) was modified on the ACC surface and continued to be conjugated with folic acid-modified and PEGylated polyamide (PAMAM) dendrimers to confer tumor targeting to the nanoparticles. After the nanoparticles were successfully intercalated into tumor cells, the ACC was hydrolyzed in the low-pH environment of lysosomes. Meanwhile, PAMAM dendritic macromolecules promoted the lysosome escape of Fe^2+^-DOX nanoparticles and the release of Fe^2+^ and DOX into the cytoplasm through the proton sponge effect. Compared with other iron death strategies that simply increase intracellular iron accumulation, the simultaneous release of DOX can induce nitrogen oxide (NOX) activation for further ROS production in addition to its cytotoxic effect, which synergizes the Fe^2+^-induced oxidative stress response, thus enhancing the iron death effect in tumor cells. In vivo anti-tumor evaluation demonstrated that ACC@DOX.Fe^2+^-CaSi-PAMAM-FA/Mpeg significantly reduced tumor volume by more than 5 times compared with the control and free DOX groups and prolonged the median survival time of the tumor-bearing mice to more than 50 days. In conclusion, the nanoformulation can effectively inhibit tumor growth in the hormonal animal model via complementary ferroptosis and chemotherapy.

Based on the chemical structures of ICG and Ce6, Yuan et al. [54] chose to use a core–shell material (HM) composed of an ACC core and a phospholipid shell as a carrier to construct a core–shell-structured nano-drug delivery system HM-I&C encapsulating ICG-Ca^2+^-Ce6 molecules (Figure 8B). The researchers creatively linked two conventional photothermal/photodynamic agents, ICG and Ce6, through Ca^2+^. Through the Förster resonance energy transfer (FRET) effect satisfied by the linkage of Ca^2+^, the ICG in the ICG-Ca^2+^-Ce6 molecule can achieve the physical quenching of Ce6 phototoxicity. When the nanoparticles enter the tumor tissue, ACC degrades under acidic conditions, and the association among Ca^2+^, ICG, and Ce6 is weakened, resulting in increased intermolecular distance, and the fluorescence property of Ce6 is restored, enabling the ce6-mediated tumor photodynamic therapy (PDT) strategy. Through the ingenious combination of ICG and Ce6, the researchers achieved a combination of photothermal therapy (PTT) and PDT in low-temperature synergistic therapy. In vivo and in vitro anti-tumor evaluation, researchers used the combination of 808/680 nm laser irradiation to compare with high-temperature PTT and PDT alone. The results showed that the efficacy of low-temperature synergistic therapy was significantly better than that of high-temperature PTT and PDT alone, and the tumor volume of tumor-bearing mice decreased significantly. Moreover, Western blotting analysis and TUNEL assay also showed that the apoptosis of tumor cells was particularly remarkable.

Natural active anti-tumor agents have an outstanding role in modulating cellular channel activity. For intracellular calcium overload, a tacit cooperation with Ca^2+^-related channels is required to achieve it, which makes many related natural active agents become potential targets for CaCO_3_-based nano-drug delivery systems. Xu and Han et al. loaded capsaicin (CAP) into CaCO_3_ nanoparticles [71]. As a natural TRPV1 channel agonist, CAP can effectively regulate the opening of the TRPV1 channel to allow Ca^2+^ to influx, which has a significant promotion effect on the influx of exogenous Ca^2+^. In combination with the release of Ca^2+^ from CaCO_3_, CaCO_3_@CAP nanoparticles effectively achieve the calcium overload effect. The results of anti-tumor evaluation in vivo showed that the tumor mass and density of tumor-bearing mice were significantly decreased, and the tumor growth was significantly inhibited. The naturalness and non-toxicity of capsaicin make the nanoparticles have good biocompatibility and good development prospects and clinical application potential.

### 4.2. Biomolecular Drug Nano-Drug Delivery Systems

Lee and Xiang et al. [78] co-loaded miR-375 and Sorafenib into CaCO_3_ nanoparticles (miR-375/Sf-LCC NPs) with a lipid coating for liver cancer therapy. The results showed that the miR-375/Sf-LCC nanoparticles had a pH-dependent release of the drug and effective cytotoxicity. And in vivo experiments demonstrated that NPs by injection increased the concentration of miR-375 and Sorafenib in tumors and prolonged the time of retention of both drugs in the tumor site compared to direct injection of the two free drugs. In addition, it was demonstrated that co-delivery of miR-375 and Sorafenib significantly inhibited Sorafenib-induced cellular autophagy, thereby reducing Sorafenib resistance in liver cancer cells. The NPs showed significant tumor volume suppression in a xenograft tumor suppression assay in nude mice, demonstrating that NPs can promote the efficient delivery of the drugs and show effective liver cancer treatment.

**Figure 9:**
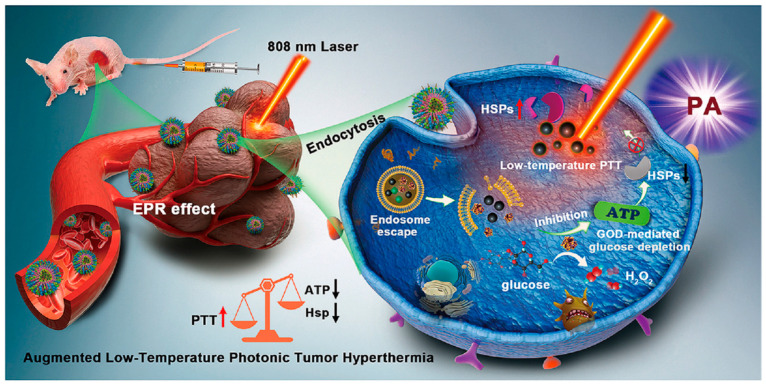
Example of biomolecular drug nano-drug delivery systems constructed based on CaCO_3_ nanoparticles. The schematic illustration shows the mechanism of a pH-sensitive Ca^2+^-based nanocatalyst, G/A@CaCO_3_-PEG, which enhances the thermotherapy-based tumor treatment by limiting ATP supply. Adopted with permission from Ref. [70]. 2023, Chenteng Lin.

Hu, Chen, and Zheng et al. [70] constructed an efficient pH-sensitive nanocatalyst (G/A@CaCO_3_-PEG) composed of CaCO_3_-loaded glucose oxidase (GOD) and 2D antimony quantum dots (AQD) (Figure 9). Afterward, the emitted GOD can effectively consume endogenous glucose and the concentration-dependent decrease in intracellular ATP levels with the increase in GOD concentration. The decrease in ATP levels also reverses the thermotolerance of tumor cells by down-regulating the expression of heat shock protein (HSP). This effect can enhance the therapeutic effect of 2D AQD-induced photothermal therapy under NIR irradiation. The results showed that G/A@CaCO_3_-PEG combined with laser irradiation induced significant apoptosis of tumor cells through mild PTT-induced HSP down-regulation and GOD-mediated glucose exhaustion and through ATP inhibition. The in vivo experiments also demonstrated that G/A@CaCO_3_-PEG in conjunction with laser effectively inhibited tumor growth in mice. This work provides an effective way to enhance photothermal-based tumor therapies by limiting ATP production in tumor cells.

Gu et al. [92] encapsulated CaCO_3_ nanoparticles containing anti-CD47 antibodies in fibrin gels and sprayed them in situ at the tumor excision area. It was demonstrated that CaCO_3_ nanoparticles could gradually lyse and free aCD47 encapsulated in specific TME and promote the revitalization of M1-type macrophages. By blocking CD47 and SIRPα interactions, macrophages and dendritic cells enhanced the phagocytosis of cancer cells and activated the natural immune system. In addition to this, the proportion of CD8+ and CD4+ T cells in the tumor was significantly increased, and the production of cytokines (including IFN-γ, IL-6, and IL-12p70) was also significantly increased after CD47@CaCO_3_@fibronectin treatment. The experimental results suggested that immunotherapeutic fibronectin gel can activate the innate and acquired immune system of the host to inhibit the local recurrence of the tumor after surgery and reduce the risk of metastatic spread.

### 4.3. Surface Mineralization Nano-Drug Delivery Systems

Covering the surface by using CaCO_3_ can effectively protect the nanoparticles to maintain stability during the process of in vivo circulation and avoid the leakage of the drug encapsulated in the nanoparticles. At the same time, CaCO_3_ can decompose in the acidic environment of the tumor, so that the nanoparticles can be specifically released. In addition, the reverse osmotic pressure of Ca^2+^ and HCO_3_^−^ [137] and gas expansion of CO_2_ [122] promote the lysosomal escape of the nanoparticles, making the drugs further enter the cytoplasm, realizing the therapeutic effect of drugs in tumor cells.

Xia and Xu et al. [138] reported mesoporous silica nanoparticles (MSNs) coated with CaCO_3_ and lipid bilayers (MSNs@CaCO_3_@liposomes). The nanoparticles can achieve continuous drug release and enhance biocompatibility in the tumor microenvironment. The CaCO_3_-mineralized layer can cap the pore channels of MSNs, allowing the drug loaded in MSNs to be transported to the tumor site without leakage and achieving a pH-triggered drug release at the target site. In the experiments, doxorubicin (DOX) was effectively loaded into MSNs. The experiments also showed that nanoparticles exhibited a delayed release of DOX at a low pH, while no drug release occurred at a normal pH.

**Figure 10:**
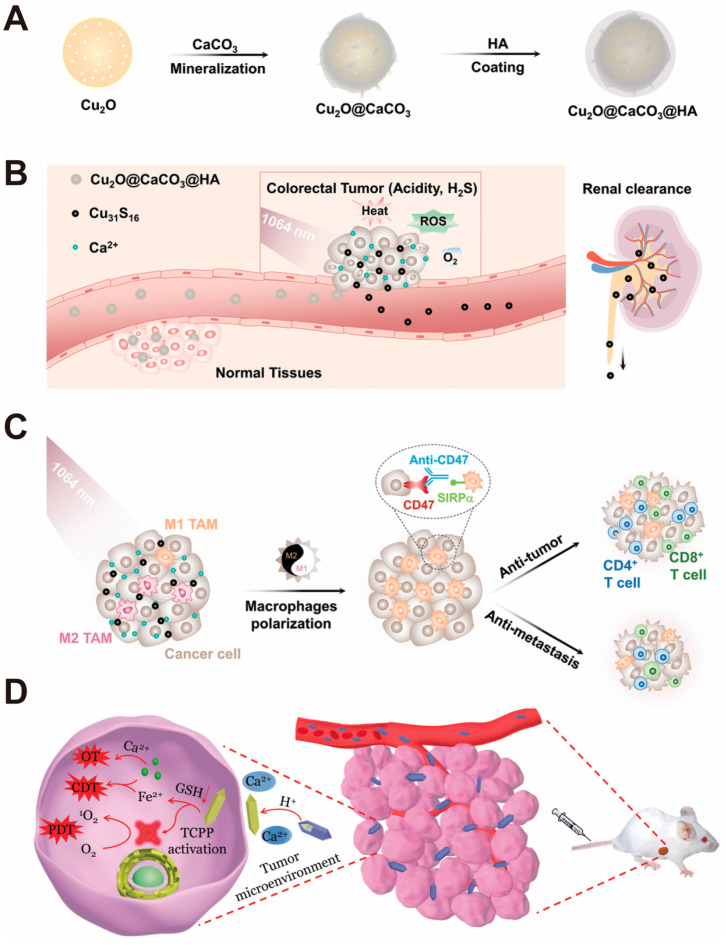
Examples of surface mineralization nano-drug delivery systems constructed based on CaCO_3_ nanoparticles. (**A**) Cu_2_O@CaCO_3_@HA (CCH) synthesis route; CCH biodegradation, anti-tumor response, and renal clearance triggered by the tumor microenvironment in colorectal cancer (**B**) and its anti-tumor immune response activated by combined CD47 blockade in the tumor microenvironment in colorectal cancer (**C**). Adopted with permission from Ref. [56]. 2023, Chenteng Lin. (**D**) Schematic illustration of the mechanism of NMOF@DHA@CaCO_3_ for programmed drug release in cancer therapy. Adopted with permission from Ref. [55]. 2023, Chenteng Lin.

Lin and Hou et al. [56] have developed a “turn-on” therapeutic nanoplatform for the delivery of targeted therapy of H_2_S-rich colorectal cancer. The researchers first prepared hollow mesoporous Cu_2_O nanoparticles with a size of ~100 nm and subsequently coated them with a CaCO_3_ shell (Cu_2_O@CaCO_3_) by in situ surface mineralization. Finally, hyaluronic acid (HA) was functionalized onto Cu_2_O@CaCO_3_, dependent on its desirable targeting ability and biocompatibility, to form Cu_2_O@CaCO_3_@HA (CCH) nanoparticles (Figure 10A–C). When the nanoparticles reach the tumor site through HA targeting of colorectal cancer, the CaCO_3_-mineralized layer breaks down in the acidic tumor microenvironment to produce Ca^2+^, causing intracellular calcium overload, thus enabling Ca^2+^-based ion interference therapy (CIT). Subsequently, when exposed Cu_2_O comes in contact with high H_2_S in colon cancer, the nanocomposite can be decomposed into ultra-small Cu_31_S_16_ nanoparticles, and the generated Cu_31_S_16_ shows outstanding photothermal properties, photocatalytic properties, and Fenton-like activity toward PTT/PDT/chemodynamic therapy (CDT).

In addition to surface mineralization of mesoporous nanoparticles, CaCO_3_ is also capable of achieving good surface wrapping for novel metal–organic framework (MOF) materials. Li and Tang et al. [55] reported that an Fe-based NMOF (nanoscale MOF) is used for the synergistic tumor treatment (Figure 10D). When the nanoplatforms reach the target site, the CaCO_3_-mineralized layer can begin to dissolve, resulting in the generation of NMOF@DHA and Ca^2+^. Subsequently, Ca^2+^ acts synergistically with Fe^3+^ in the NMOF@DHA structure, DHA, and the photosensitizer TCPP through Fe^2+^-DHA-mediated chemodynamic therapy, Ca^2+^-DHA-mediated tumor therapy (OT), and TCPP-mediated PDT showing a triple synergistic therapeutic effect and exhibiting high therapeutic efficiency, capable of achieving complete tumor ablation.

Zhang et al. [44] constructed a lysosome-targeted nanoparticle (LYS-NP) by combining a mineralized MOF with a targeted inducer (CD63-aptamer) to enhance the anti-tumor quality of T cells. CaCO_3_ was used to induce the mineralization of Zn-based MOF, conferring good biocompatibility and acid degradation to the nanoparticles, while also improving the stability of the composite-encapsulated therapeutic proteins. Ca^2+^ produced by the CaCO_3_ mineralization layer can also enhance the function of perforin and granzyme B. Upon entry into the body, LYS-NPs are targeted to tumor cell lysosomes with the aid of CD63-aptamer. Under lysosomal acidic conditions, LYS-NPs are degraded and the degradation products, perforin, granzyme B, and Ca^2+^, are released into the lysosomes. Upon activation of the T cell receptor (TCR) by the major histocompatibility complex (MHC) of tumor cells, adoptive T cell vectors (ATVs) activate the under autonomous controlled release of perforin, granzyme B and Ca^2+^ into lysosomes to treat tumors. This study constructed a “super cytotoxic T lymphocyte” that targets tumors and releases cytotoxic proteins, opening up a new strategy for immunotherapy of solid tumors.

## 5. Conclusions and Perspective

In recent years, CaCO_3_-based nano-drug delivery systems have been widely used in the field of oncology diagnosis and treatment. This enables CaCO_3_-based nano-drug delivery systems as carriers or shell structures to maintain high structural integrity during in vivo circulation to ensure the least possible leakage of the drug molecules or nanostructures encapsulated within them, minimizing the systemic toxic effects of the drugs. Meanwhile, in the low-pH environment of tumor tissues, CaCO_3_ can rapidly respond to degradation and release drug molecules and nanostructures in a targeted manner to achieve targeted drug accumulation and release in tumor sites. In addition, the degradation of CaCO_3_ can also generate large amounts of Ca^2+^ and CO_2_, the former of which has a key position in the regulation of cell metabolism, giving it special therapeutic effects in tumor therapy, such as calcium ion interference therapy. The rapid intracellular production of CO_2_ leads to rapid gas expansion, which promotes lysosomal escape and the release of drugs and nanoparticles and plays a key role in tumor diagnosis as an enhancer for magnetic resonance imaging and ultrasound development. Therefore, with excellent biocompatibility, sensitive pH degradation, high Ca^2+^ content, and CO_2_ generation properties, CaCO_3_-based nano-drug delivery systems have shown significant advantages in drug delivery carriers, tumor therapy, and tumor diagnostics in vivo and in vitro.

At the same time, CaCO_3_-based nano-drug delivery systems, due to their good biocompatibility, are able to avoid many of the toxic side effects of other traditional inorganic nanomaterials when used as drug delivery carriers in vivo, which include long-term potential toxicity and low clearance in vivo. This advantage also implies that CaCO_3_-based nano-drug delivery systems are highly promising for clinical translation and are expected to become an advancing nanomedicine for tumor diagnosis and treatment.

However, it should also be noted that CaCO_3_ has variable crystal types, which may undergo reversible/irreversible transitions between various crystal types under different external conditions and environments. This leads to the need to pay particular attention to the choice of reaction conditions and the control of nanoparticle dissolution–crystallization nucleation rates when synthesizing CaCO_3_ nanoparticles, so that the CaCO_3_-based nano-drug delivery system is suitable for drug delivery. The long-range disorder and instability of amorphous calcium carbonate (ACC) make it promising for a wide range of applications in drug loading and delivery as a highly acid-responsive material, but its structure remains mysterious. Researchers have now studied the clustering and crystalline nucleation of ACC in some depth and discovered the key role played by water molecules, which is of great significance for the further application of ACC in the future.

Therefore, CaCO_3_-based nano-drug delivery systems with good biocompatibility, sensitive pH degradation, high Ca^2+^ content, and CO_2_ generation characteristics show significant advantages in the field of tumor diagnosis and therapy as carriers for in vivo drug delivery. It is believed that in the future, with the deepening research on CaCO_3_/Ca^2+^ mode of action and tumor therapeutic methods, it is expected to become an advancing nanomedicine for clinical tumor diagnosis and treatment.

## Figures and Tables

**Table 1:** Representative CaCO_3_-based nano-drug delivery systems prepared by chemical precipitation method.

CaCO_3_-Based Nano-Drug Delivery System	Sources of Ca^2+^ and CO_3_^2−^	Modifier/Template/Core	Particle Sizes	Reference
HMME/MCC-HA	CaCl_2_ and Na_2_CO_3_ solution	soluble starch solution	# 264.5 ± 4.7 nm	[33]
Zeb-aPD1-NPs-Gel	CaCl_2_ and Na_2_CO_3_ solution	PEG-P(Glu)	#* ~100 nm (aPD1-NPs)	[34]
CM@CaCO_3_@SAF NPs	CaCl_2_ and Na_2_CO_3_ solution	SAF NPs@DOX	#* 150~160 nm (CaCO_3_@SAF NPs) #* ~200 nm (CM@CaCO_3_@SAF NPs)	[35]
SH@FP@CaCO_3_ vaccine	CaCl_2_ and Na_2_CO_3_ solution	silk fibroin solution + acetic acid solution	—	[36]
ACC@Cu_2_O-TPP NCs	anhydrous CaCl_2_ and Dimethyl carbonate (DMC)	—	# 93.38 nm (ACC NPs) # 210.398 nm (ACC@Cu_2_O-TPP NCs)	[37]
HA-DOX/CaCO_3_	(Ca(NO_3_)_2_·4H_2_O and Na_2_CO_3_ solution	Low-molecular-weight sodium hyaluronate	# 88.5 nm	[38]
Cu/SOD-MNPs	CaCl_2_ and Na_2_CO_3_ solution	PEG-PAsp	* 220 nm	[39]
Au@CaCO_3_ NPs	CaCl_2_ and Na_2_CO_3_ solution	AuNPs	* 32 nm	[40]
HOCN (OVA@CaCO_3_)	CaCl_2_ and Na_2_CO_3_ solution	Ovalbumin (OVA)	# 250 nm	[41]
^Ca^NP/DOX	(Ca(NO_3_)_2_ and Na_2_CO_3_ solution	Mpeg-b-PGA	* 150.3 ± 8.6 nm (TEM) # 103.0 ± 7.5 nm (DLS)	[42]
CaCO_3_@COF-BODIPY-2I@GAG	CaCl_2_ and NH_4_HCO_3_ solution	COF-BODIPY-2I	#* 180.4 nm (CaCO_3_@COF-BODIPY-2I) #* 319.4 nm (CaCO_3_@COF-BODIPY-2I@GAG)	[43]
LYS-NPs	CaCl_2_ and Na_2_CO_3_ solution	ZIF-8 NPs	# 270.6 nm	[44]
MNCa^⊕^	CaCl_2_ and Na_2_CO_3_ solution	MN^⊕^	* ~120 nm	[45]
OVA@NP	CaCl_2_ and Na_2_CO_3_ solution	Ovalbumin (OVA)	* ~500 nm (OVA@NP) * ~30 nm (CaCO_3_)	[46]
PDA/BSA/CaCO_3_ Hybrid Particles	CaCl_2_·2H_2_O and Na_2_CO_3_·2H_2_O solution	dopamine hydrochloride (PDA) and BSA	# 572 nm	[47]

Note: # hydrodynamic particle sizes, * particle sizes measured by Transmission Electron Microscope, #* particle sizes measured by both methods. HMME: hematoporphyrin monomethyl ether, MCC: mesoporous calcium carbonate, HA: hyaluronic acid, Zeb: zebularine, aPD1: anti-PD1 antibody, Gel: hydrogel, PEG-P(Glu): poly(ethylene glycol)-poly(glutamic acid), CM: cell membrane, SAF NPs: single-atom iron nanoparticles, DOX: doxorubicin, SH: silk fibroin hydrogel, FP: 4T1 cells-DC fusion cells, ACC: amorphous calcium carbonate, TPP: triphenylphosphine, NCs: nanocages, SOD: superoxide dismutase, MNPs: mineralized nanoparticles, PEG-PAsp: poly(ethylene glycol)-b-poly(l-aspartic acid), AuNPs: gold nanoparticles, HOCN: honeycomb calcium carbonate nanoparticle, OVA: ovalbumin, Mpeg-b-PGA: methoxy poly(ethylene glycol)-block-poly(l-glutamic acid), COF: covalent organic framework, BODIPY-2I: BODIPY-2I photosensitizer, GAG: glycosaminoglycan, LYS-NP: lysosome-targeting nanoparticle, ZIF-8: a metal–organic framework made of Zn^2+^ ions connected by 2-methylimidazole bridging units, MNCa⊕: positively-charged Fe_3_O_4_@CaCO_3_, MN⊕: positive-charged magnetic nanoparticle, OVA@NP: ovalbumin@CaCO_3_ nanoparticle, PDA: polydopamine, BSA: bovine serum albumin.

**Table 2:** Representative CaCO_3_-based nano-drug delivery systems prepared by gas diffusion method.

CaCO_3_-Based Nano-Drug Delivery System	Sources of Ca^2+^ and CO_3_^2−^	Conditions (Medium and Temperature and Time)	Particle Sizes	Reference
MS/ACC–DOX NPs	CaCl_2_ and (NH_4_)_2_CO_3_	ethanol and 25 °C and 2–3 days	#* ~80 nm (ACC–DOX NPs) #* ~100 nm (MS/ACC–DOX NPs)	[50]
CaCO_3_-TPZ@GOD@HA (AC-TGH) NPs	CaCl_2_ and NH_4_HCO_3_	anhydrous ethanol and 30 °C and 60 h	* ~80 nm (AC-T NPs) # 161 nm (AC-TGH NPs)	[51]
TPZ@CaCO3-PDA-ICG-TPGS/TPGS-RGD nanoparticles (ICG-PDA-TPZ NPs)	CaCl_2_·6H_2_O and NH_4_HCO_3_	ethanol and 24 h	# 104.7 ± 1.3 nm (TPZ@CaCO3 nanoparticles) # 178.5 ± 1.8 nm (TPZ@CaCO3-PDA-ICG-TPGS/TPGS-RGD nanoparticles)	[52]
PL/ACC-DOX&ICG	CaCl_2_ and (NH_4_)_2_CO_3_	anhydrous ethanol and 30 °C and 48 h	* ~80 nm (ACC-DOX&ICG) * ~100 nm (PL/ACC-DOX&ICG)	[53]
HM-I&C	CaCl_2_ and (NH_4_)_2_CO_3_	anhydrous ethanol and 25 °C and 2–3 days	# ~100 nm	[54]
NMOF@DHA@CaCO_3_	CaCl_2_·2H_2_O and NH_4_HCO_3_	ethanol and room temperature and 24 h	# 382 ± 23 nm in length # 182 ± 37 nm in width	[55]
Cu_2_O@CaCO_3_@HA (CCH)	CaCl_2_ and NH_4_HCO_3_	ethanol and room temperature and 8 h	# ~180 nm (average hydrodynamic diameter) * 167.6 nm (physical particle size)	[56]
BSO-TCPP-Fe@CaCO_3_-PEG	CaCl_2_·2H_2_O and NH_4_HCO_3_	ethanol and 24 h	# 193.4 ± 2.4 nm (DLS) * 125.2 ± 7.7 nm (TEM)	[57]
DOX/GA–Fe@CaCO_3_-PEG	CaCl_2_·2H_2_O and NH_4_HCO_3_	ethanol and 24 h	* ~106.3 nm (CaCO_3_ NPs) * ~109.2 nm (GA–Fe@CaCO_3_)	[58]
CaCO_3_@IDOi@PEG@PEI@CpG (CaIPC) nanoparticles	CaCl_2_·2H_2_O and NH_4_HCO_3_	ethanol and 24 h	* 55–65 nm	[59]
IMQ@ACC(Mn)–ICG/PEG nanoparticles	CaCl_2_ and NH_4_HCO_3_	ethanol and room temperature and 2 days	* ~43 nm (ACC(Mn)-ICG NPs) # ~72.4 nm (ACC(Mn)–ICG/PEG NPs)	[60]
M@CaCO_3_@KAE nanoparticles	CaCl_2_ and NH_4_HCO_3_	ethanol and 45 °C and 4 d	#* ~100 nm	[61]
ACC@Fe^2+^/BLM-CaSi-GP	CaCl_2_ and NH_4_HCO_3_	anhydrous ethanol and 30 °C and 36 h	* 78.8 nm (ACC@Fe^2+^/BLM) # ~100 nm (ACC@Fe^2+^/BLM-CaSi)	[62]
Mn/CaCO_3_@PL/SLC NPs	CaCl_2_ and NH_4_HCO_3_	anhydrous ethanol and 40 °C and 24 h	* ~138 nm (CaCO_3_ NPs) #* ~183 nm (Mn/CaCO_3_@PL/SLC NPs)	[63]
^PEG^CaCUR	CaCl_2_·2H_2_O and NH_4_HCO_3_	ethanol and 40 °C and 10 h	* ~140 nm	[64]
^PEG^CaNM_CUR+CDDP_	CaCl_2_·2H_2_O and NH_4_HCO_3_	ethanol and 40 °C and 18 h	CaNM_CUR+CDDP_ 211 nm (TEM)/252 nm (DLS) CaNM_CUR_ 198 nm (TEM)/200 nm (DLS) CaNM_CDDP_ 127 nm (TEM)/130 nm (DLS) CaNM 121 nm (TEM)/127 nm (DLS)	[65]
Mn:CaCO_3_-DEX	CaCl_2_·2H_2_O and NH_4_HCO_3_	ethanol and room temperature and 8 h	* 150.4 ± 20.2 nm (CaCO_3_-DEX)	[66]
Ca@H NPs	CaCl_2_·2H_2_O and NH_4_HCO_3_	ethanol and 48 h	~200 nm (TEM) 216.2 ± 46.09 nm (DLS)	[67]
DiR-DOX-Gd@pCaCO_3_-PEG	CaCl_2_·2H_2_O and NH_4_HCO_3_	anhydrous ethanol and 40 °C and 24 h	# 156.5 nm (DiR-DOX@pCaCO_3_-PEG)	[68]
IrCOOH–CaCO_3_@PEG	CaCl_2_·2H_2_O and NH_4_HCO_3_	ethanol and 30 °C and 24 h	# 126.73 ± 0.65 nm (CaCO_3_) # 168.76 ± 3.73 nm (IrCOOH–CaCO_3_) # 188.75 ± 3.79 nm (IrCOOH–CaCO_3_@PEG)	[69]
G/A@CaCO_3_-PEG	CaCl_2_·2H_2_O and NH_4_HCO_3_	anhydrous ethanol and 40 °C and 12 h	* 108 nm (CaCO_3_ NPs) # 114 ± 4.8 nm (G/A@CaCO_3_) # 126 ± 6.3 nm (G/A@CaCO_3_-PEG)	[70]
CaCO_3_@CAP-PEG nanoparticle	CaCl_2_·2H_2_O and NH_4_HCO_3_	ethanol and 40 °C and 24 h	CaCO_3_@CAP ~40 nm (TEM)/45 nm (DLS)	[71]
O_2_-FeCOF@CaCO_3_@FA (OFCCF)	CaCl_2_·2H_2_O and NH_4_HCO_3_	ethanol and 40 °C and 8 h	* 200~250 nm	[72]
CaNMs	CaCl_2_·2H_2_O and NH_4_HCO_3_	ethanol and 40 °C and 10 h	* ~200 nm	[73]

Note: # hydrodynamic particle sizes, * particle sizes measured by Transmission Electron Microscope, #* particle sizes measured by both methods. MS: monostearate, TPZ: tirapazamine, GOD: glucose oxidase, AC-T: TPZ@CaCO_3_, AC-TGH: TPZ@CaCO_3_@GOD@HA, ICG: indocyanine green, TPGS: D-α-tocopheryl polyethylene glycol (PEG) 1000 succinate, RGD: c(RGDfK) peptide, PL: phospholipid, HM: hybrid materials, I&C: indocyanine green (ICG) & chlorins e6 (Ce6), NMOF: nanoscale metal–organic framework, DHA: dihydroartemisinin, BSO: L-buthionine sulfoximine, TCPP: meso-tetra-(4-carboxyphenyl)porphine, PEG: polyethylene glycol, GA: gallic acid, IDOi: indoleamine 2,3-Dioxygenase inhibitor, PEI: polyethyleneimine, CpG: cytosine-phosphate-guanosine, IMQ: imiquimod, ACC(Mn): Mn^2+^-doped amorphous calcium carbonate, M: cancer cell membrane, KAE: kaempferol-3-O-rutinoside, BLM: bleomycin, CaSi: an additional layer of SiO_2_-CaCO_3_, GP: electrospun gelatin/polycaprolactone, PL: palmitoyl ascorbate-liposome, SLC: carbonic anhydrase inhibitor SLC-0111, CUR: curcumin, CDDP: cisplatin, Mn:CaCO_3_: Mn-doped calcium carbonate, DEX: dexamethasone phosphate, Ca@H NPs: hematoporphyrin monomethyl ether-loaded CaCO_3_ nanoparticles, DiR: 1,1′-dioctadecyl-3,3,3′,3′-tetramethylindotricarbocyanine iodide, Gd: gadolinium, pCaCO_3_: calcium carbonate-polydopamine, IrCOOH: Ir(III) complexes, G/A: glucose oxidase (GOD)/2D antimonene quantum dots (AQDs), CAP: capsaicin, FeCOF: Fe^2+^-doped covalent organic framework, O_2_-FeCOF: O_2_-storaged Fe^2+^-doped COF, FA: folic acid, CaNMs: Ca^2+^ nanomodulators.

**Table 3:** Representative CaCO_3_-based nano-drug delivery systems prepared by microemulsion method.

CaCO_3_-Based Nano-Drug Delivery System	Sources of Ca^2+^ and CO_3_^2−^	Emulsion Principle	Water Phase and Oil Phase	Particle Sizes	Reference
mCNPs	CaCl_2_ and Na_2_CO_3_	W_1_/O/W_2_ double emulsion method	W_1_ phase: CaCl_2_ (27.7% *w*/*v*) Oil phase: PLG (5% *w*/*v*, Dichloromethane) W_2_ phase: PVA (4% *w*/*v*) + Na_2_CO_3_ (1.06% *w*/*v*)	# ~200 nm	[77]
miR-375/Sf-LCC NPs	CaCl_2_ and Na_2_CO_3_	W/O reverse microemulsion method	Water phase: Water Oil phase: Cyclohexane/Igepal CO-520 (71:29, *v*/*v*)	# 100.7 ± 12.1 nm	[78]
lipid/CaCO_3_/curcumin (LCC)	CaCl_2_ and Na_2_CO_3_	W/O reverse microemulsion method	Water phase: Water Oil phase: Dichloromethane	# 155.3 nm	[79]
CDDP/OA-LCC NPs	CaCl_2_ and Na_2_CO_3_	W/O reverse microemulsion method	Water phase: Water Oil phase: Cyclohexane/Igepal CO-520 (71:29, *v*/*v*)	* 206 ± 15 nm	[80]
HDL/CC/DOX NPs	CaCl_2_ and Na_2_CO_3_	W/O reverse microemulsion method	Water phase: Water Oil phase: Cyclohexane/Triton X-100/n-hexanol (*v*/*v*: 70/20/10)	# 68.2 ± 3.9 nm	[81]
DOX@CaCO_3_ NPs	CaCl_2_ and Na_2_CO_3_	W/O reverse microemulsion method	Water phase: Water Oil phase: n-hexane + n-butyl alcohol + CTAB	# 70.6 nm ± 0.9 nm	[82]
DNCaNPs	CaCl_2_ and NaHCO_3_	W_1_/O/W_2_ double emulsion method	W_1_ phase: Water Oil phase: DCM (PLGA + PLGA-PEG + Anlg919) W_2_ phase: PVA (1 wt%)	* ~100 nm	[83]
CDDP/OA-LCC NPs	CaCl_2_ and Na_2_CO_3_	W/O reverse microemulsion method	Water phase: Water Oil phase: Cyclohexane/Igepal CO-520 (71:29, *v*/*v*)	# 217 ± 20 nm	[84]
ECCaNPs	CaCl_2_ and NaHCO_3_	W_1_/O/W_2_ double emulsion method	W_1_ phase: Water Oil phase: DCM (PLGA + PLGA-PEG + Erlotinib) W_2_ phase: PVA (1 wt%)	#* ~100 nm	[85]

Note: # hydrodynamic particle sizes, * particle sizes measured by Transmission Electron Microscope, #* particle sizes measured by both methods. MCNPs: poly(D,L-lactide-co-glycolide) (PLG) nanoparticles containing mineralized calcium carbonate, Sf: sorafenib, LCC NPs: calcium carbonate nanoparticles with lipid coating, CDDP: cisplatin, OA: oleanolic acid, HDL: high-density lipoprotein, CC: calcium carbonate, DNCaNPs: doxorubicin/alkylated NLG919-loaded CaCO_3_ nanoparticles, ECCaNPs: erlotinib-chlorin e6-loaded CaCO_3_ nanoparticles.

**Table 4:** Summary of the basic characteristics of different types of CaCO_3_-based nano-drug delivery systems for tumor diagnosis and treatment.

CaCO_3_-Based Nano-Drug Delivery System	Types of Nano-Drug Delivery Systems	Particle Loading	Function of CaCO_3_	Therapeutic Strategy	Reference
Zeb-aPD1-NPs-Gel	Biomolecular Drug	Anti-PD1 antibody (aPD1)	· pH-responsive drug carrier	· Drug therapy · Immunotherapy	[34]
aCD47@CaCO_3_	Biomolecular Drug	Anti-CD47 antibody (aPD47)	· A release reservoir of drug · A proton scavenger	· Immunotherapy	[92]
CM@CaCO_3_@SAF NPs	Surface Mineralization	SAF NPs@DOX	· In situ mineralized therapeutic agent · Calcium ion supplier · pH-responsive drug carrier	· Calcium ion interference therapy · Chemotherapy · Chemodynamic therapy	[35]
Au@CaCO_3_ NPs	Surface Mineralization	AuNPs	· Encapsulating agent	· Therapy based on macrophage activation	[40]
LYS-NPs	Surface Mineralization	ZIF-8 NPs	· In situ mineralizer · Calcium ion supplier	· T cell immunotherapy	[44]
MNCa⊕	Surface Mineralization	positive-charged Fe_3_O_4_ nanoparticle (MN⊕)	· pH-responsive mineralizer	· Circulating tumor cell capture agent	[45]
DSA/CC-DOX NPs	Small-Molecule Drug	Doxorubicin (DOX)	· pH-responsive drug carrier	· Chemotherapy	[122]
Fe_3_O_4_@PDA@CaCO_3_/ICG (FPCI) NPs	Surface Mineralization	Fe_3_O_4_@PDA and ICG	· pH-responsive drug carrier	· Photothermal therapy · Photodynamic therapy	[123]
GNS@CaCO_3_/Ce6-NK	Small-Molecule Drug and Surface Mineralization	Chlorin e6 (Ce6) and Gold nanostars (GNS)	· pH-responsive mineralizer	· Fluorescence imaging and Photoacoustic imaging · Photothermal/photodynamic therapy · Immunotherapy	[124]
CaCO_3_-DOX NPs	Small-Molecule Drug	Doxorubicin (DOX)	· CO_2_-releasing agent · pH-responsive drug carrier	· Ultrasound imaging · Fluorescence imaging · Chemotherapy	[120]
BSA/AIEgen@CaCO_3_	Small-Molecule Drug	1-methyl-4-(4-(1,2,2-triphenylvinyl) styryl) quinolinium iodide (TPE-Qu+)	· pH-responsive mineralizer	· Photodynamic therapy	[125]
Bi_2_S_3_@CaCO_3_ NRs	Surface Mineralization	Bi_2_S_3_ nanorods	· pH-responsive mineralizer	· Photothermal therapy	[126]
PGP/CaCO_3_@IR820/DTX-HA	Small-Molecule Drug and Surface Mineralization	IR820 and Docetaxel (DTX) and Pentagonal gold prisms (PGPs)	· pH-responsive drug carrier	· Photothermal therapy · Photodynamic therapy · Chemotherapy	[127]
Fe@CaCO_3_/ICG	Small-Molecule Drug and Surface Mineralization	Fe_3_O_4_ NPs and IR820 and ICG	· pH-responsive mineralizer	· Photothermal therapy · Photodynamic therapy	[128]
Alg-CaCO_3_-PDA-PGED (ACDP)	Surface Mineralization	Alginate (Alg) micelles	· pH-responsive gene carrier	· Mild hyperthermia-enhanced gene therapy · Ultrasound imaging · Photoacoustic imaging	[129]
LMGC NPs	Surface Mineralization	LMG NPs	· pH-responsive mineralizer · Calcium ion supplier	· ATP generation inhibition · Photothermal therapy	[130]
CaCO_3_-TPZ@GOD@HA (AC-TGH) NPs	Small-Molecule and Biomolecular Drug	Glucose oxidase (GOD) and Tirapazamine (TPZ)	· pH-responsive drug carrier	· Tumor starvation therapy · Chemotherapy	[51]
TPZ@CaCO_3_-PDA-ICG-TPGS/TPGS-RGD (ICG-PDA-TPZ NPs)	Small-Molecule Drug	Tirapazamine (TPZ)	· pH-responsive drug carrier	· Photothermal therapy · Photodynamic therapy · Chemotherapy	[52]
PL/ACC-DOX&ICG	Small-Molecule Drug	Indocyanine green (ICG) and Doxorubicin (DOX)	· pH-responsive drug carrier	· Near-infrared (NIR) imaging · Photothermal therapy · Chemotherapy	[53]
NMOF@DHA@CaCO_3_	Surface Mineralization	NMOF@DHA	· pH-responsive mineralizer · Calcium ion supplier	· Ca^2+^-DHA-mediated oncosis therapy · Photodynamic therapy	[55]
Cu_2_O@CaCO_3_@HA (CCH)	Surface Mineralization	Hollow mesoporous Cu_2_O	· pH-responsive mineralizer · Calcium ion supplier	· Photothermal therapy · Photodynamic therapy · Chemodynamic therapy · Calcium-overload-mediated therapy · Immunotherapy	[56]
CaCO_3_@IDOi@PEG@PEI@CpG (CaIPC) nanoparticles	Small-Molecule and Biomolecular Drug	Cytosine-phosphate-guanosine oligonucleotides (CpG ODNs) and IDO inhibitor INCB24360 (IDOi)	· pH-responsive drug carrier · Calcium ion supplier	· T cell immunotherapy	[59]
IMQ@ACC(Mn)–ICG/PEG nanoparticles	Small-Molecule Drug	Indocyanine green (ICG) and Imiquimod (IMQ)	· pH-responsive drug carrier	· Photoimmunotherapy	[60]
M@CaCO_3_@KAE nanoparticles	Small-Molecule Drug	Kaempferol-3-O-rutinoside (KAE)	· pH-responsive drug carrier · Calcium ion supplier	· Calcium overload tumor therapy	[61]
ACC@Fe^2+^/BLM-CaSi-GP	Small-Molecule Drug	Fe^2+^ and Bleomycin (BLM)	· pH-responsive drug carrier · Proton scavengers	· Postoperative management of melanoma	[62]
Ca@H NPs	Small-Molecule Drug	Hematoporphyrin monomethyl ether (HMME)	· pH-responsive drug carrier · CO_2_-releasing agent	· High-intensity focused ultrasound · Sonodynamic therapy · Photoacoustic (PA) imaging	[67]
IrCOOH–CaCO_3_@PEG	Small-Molecule Drug	IrCOOH (Ir(III) complexs)	· pH-responsive drug carrier · Calcium ion supplier	· Calcium overload tumor therapy · Two-photon photodynamic therapy	[69]
G/A@CaCO_3_-PEG	Biomolecular Drug and Surface Mineralization	2D antimonene quantum dots (AQDs) and glucose oxidase (GOD)	· pH-responsive drug carrier	· Low-temperature photothermal therapy	[70]
CaCO_3_@CAP-PEG nanoparticle	Small-Molecule Drug	Capsaicin	· pH-responsive drug carrier · Calcium ion supplier	· Calcium overload tumor therapy	[71]
O_2_-FeCOF@CaCO_3_@FA (OFCCF)	Surface Mineralization	FeCOF	· pH-responsive mineralizer · Calcium ion supplier	· Photodynamic therapy · Calcium overload tumor therapy	[72]
^PGF^CaCO_3_-PEG	Small-Molecule Drug	Gallic acid (GA) and Fe^2+^ and Pt(IV)-SA	· pH-responsive drug carrier	· Ferroptosis · Chemotherapy	[131]
miR-375/Sf-LCC NPs	Small-Molecule and Biomolecular Drug	miR-375 and Sorafenib	· pH-responsive gene carrier · pH-responsive drug carrier	· Gene therapy · Chemotherapy	[78]
CDDP/OA-LCC NPs	Small-Molecule Drug	Cisplatin and oleanolic acid	· pH-responsive drug carrier	· Combination chemotherapy	[80]
HDL/CC/DOX NPs	Small-Molecule Drug	Doxorubicin (DOX)	· pH-responsive drug carrier	· Chemotherapy	[81]
DOX@CaCO_3_ NPs	Small-Molecule Drug	Doxorubicin (DOX)	· pH-responsive drug carrier	· Chemotherapy · Starving tumor therapy	[82]
DNCaNPs	Small-Molecule Drug	Doxorubicin (DOX) and alkylated NLG919 (Anlg919)	· pH-responsive drug carrier	· Chemo-immunotherapy	[83]
ECCaNPs	Small-Molecule Drug	Erlotinib and chlorin e6 (Ce6)	· pH-responsive drug carrier	· Chemotherapy · Photodynamic therapy	[85]

DSA: sodium alginate, AIEgen: a mitochondria-specific aggregation-induced emission (AIE)-active photosensitizer of 1-methyl-4-(4-(1,2,2-triphenylvinyl)styryl)quinolinium iodide (TPE-Qu+), Bi_2_S_3_@CaCO_3_ NRs: CaCO_3_-encapsulated Bi_2_S_3_ nanorods, PGP: pentagonal gold prisms, IR820: photosensitizer IR820, DTX: docetaxel, Alg: polysaccharide sodium alginate, PGED: ethylenediamine-functionalized poly(glycidyl methacrylate), LMGC NPs: liquid metal@Glucose oxidase@CaCO_3_ nanoparticles, PGF: (Pt(IV)-SA) and metal-polyphenol coordination polymer composed of gallic acid (GA) and Fe^2+^.

## References

[1] Ferlay J., Colombet M., Soerjomataram I., Parkin D.M., Piñeros M., Znaor A., Bray F. (2021). Cancer statistics for the year 2020: An overview. Int. J. Cancer.

[2] Fathi N., Rashidi G., Khodadadi A., Shahi S., Sharifi S. (2018). STAT3 and apoptosis challenges in cancer. Int. J. Biol. Macromol..

[3] Lu J., Liu X., Liao Y.-P., Wang X., Ahmed A., Jiang W., Ji Y., Meng H., Nel A.E. (2021). Retraction of “Breast Cancer Chemo-immunotherapy through Liposomal Delivery of an Immunogenic Cell Death Stimulus Plus Interference in the IDO-1 Pathway”. ACS Nano.

[4] Shen S., Li H.J., Chen K.G., Wang Y.C., Yang X.Z., Lian Z.X., Du J.Z., Wang J. (2017). Spatial Targeting of Tumor-Associated Macrophages and Tumor Cells with a pH-Sensitive Cluster Nanocarrier for Cancer Chemoimmunotherapy. Nano Lett..

[5] National Nanotechnology Initiative (NNI) (2024). National Nanotechnology Initiative. https://www.nano.gov/.

[6] Mu W., Chu Q., Liu Y., Zhang N. (2020). A Review on Nano-Based Drug Delivery System for Cancer Chemoimmunotherapy. Nano-Micro Lett..

[7] Patra J.K., Das G., Fraceto L.F., Campos E.V.R., Rodriguez-Torres M.D.P., Acosta-Torres L.S., Diaz-Torres L.A., Grillo R., Swamy M.K., Sharma S. (2018). Nano based drug delivery systems: Recent developments and future prospects. J. Nanobiotechnol..

[8] Nichols J.W., Bae Y.H. (2014). EPR: Evidence and fallacy. J. Control. Release.

[9] Dreher M.R., Liu W., Michelich C.R., Dewhirst M.W., Yuan F., Chilkoti A. (2006). Tumor Vascular Permeability, Accumulation, and Penetration of Macromolecular Drug Carriers. J. Natl. Cancer Inst..

[10] Fadeel B., Garcia-Bennett A.E. (2010). Better safe than sorry: Understanding the toxicological properties of inorganic nanoparticles manufactured for biomedical applications. Adv. Drug Deliv. Rev..

[11] Kumar M.N.R. (2000). Nano and microparticles as controlled drug delivery devices. J. Pharm. Pharm. Sci..

[12] Paciotti G.F., Kingston D.G.I., Tamarkin L. (2006). Colloidal gold nanoparticles: A novel nanoparticle platform for developing multifunctional tumor-targeted drug delivery vectors. Drug Dev. Res..

[13] Anglin E.J., Cheng L., Freeman W.R., Sailor M.J. (2008). Porous silicon in drug delivery devices and materials. Adv. Drug Deliv. Rev..

[14] Jain T.K., Morales M.A., Sahoo S.K., Leslie-Pelecky D.L., Labhasetwar V. (2005). Iron Oxide Nanoparticles for Sustained Delivery of Anticancer Agents. Mol. Pharm..

[15] Ginebra M., Traykova T., Planell J. (2006). Calcium phosphate cements as bone drug delivery systems: A review. J. Control. Release.

[16] Avaro J.T., Ruiz-Agudo C., Landwehr E., Hauser K., Gebauer D. (2019). Impurity-free amorphous calcium carbonate, a preferential material for pharmaceutical and medical applications. Eur. J. Miner..

[17] Boyjoo Y., Pareek V.K., Liu J. (2014). Synthesis of micro and nano-sized calcium carbonate particles and their applications. J. Mater. Chem. A.

[18] Cartwright J.H.E., Checa A.G., Gale J.D., Gebauer D., Sainz-Díaz C.I. (2012). Calcium carbonate polyamorphism and its role in biomineralization: How many amorphous calcium carbonates are there?. Angew. Chem. Int. Ed..

[19] Liu Y., Cui Y., Guo R. (2012). Amphiphilic Phosphoprotein-Controlled Formation of Amorphous Calcium Carbonate with Hierarchical Superstructure. Langmuir.

[20] Saharay M., Yazaydin A.O., Kirkpatrick R.J. (2013). Dehydration-Induced Amorphous Phases of Calcium Carbonate. J. Phys. Chem. B.

[21] Dizaj S.M., Barzegar-Jalali M., Zarrintan M.H., Adibkia K., Lotfipour F. (2015). Calcium carbonate nanoparticles as cancer drug delivery system. Expert Opin. Drug Deliv..

[22] Dong Z., Liu Y., Wang C., Hao Y., Fan Q., Yang Z., Li Q., Feng L., Liu Z. (2023). Tumor Microenvironment Modulating CaCO_3_-Based Colloidosomal Microreactors Can Generally Reinforce Cancer Immunotherapy. Adv. Mater..

[23] Huang J., He J., Wang J., Li Y., Xu Z., Zhang L., Kang Y., Xue P. (2023). Calcium carbonate-actuated ion homeostasis perturbator for oxidative damage-augmented Ca2+/Mg2+ interference therapy. Biomaterials.

[24] Dong Z., Feng L., Zhu W., Sun X., Gao M., Zhao H., Chao Y., Liu Z. (2016). CaCO_3_ nanoparticles as an ultra-sensitive tumor-pH-responsive nanoplatform enabling real-time drug release monitoring and cancer combination therapy. Biomaterials.

[25] Som A., Raliya R., Tian L., Akers W., Ippolito J.E., Singamaneni S., Biswas P., Achilefu S. (2016). Monodispersed calcium carbonate nanoparticles modulate local pH and inhibit tumor growth in vivo. Nanoscale.

[26] Dizaj S.M., Sharifi S., Ahmadian E., Eftekhari A., Adibkia K., Lotfipour F. (2019). An update on calcium carbonate nanoparticles as cancer drug/gene delivery system. Expert Opin. Drug Deliv..

[27] Min K.H., Min H.S., Lee H.J., Park D.J., Yhee J.Y., Kim K., Kwon I.C., Jeong S.Y., Silvestre O.F., Chen X. (2015). pH-controlled gas-generating mineralized nanoparticles: A theranostic agent for ultrasound imaging and therapy of cancers. ACS Nano.

[28] Zhang M., Song R., Liu Y., Yi Z., Meng X., Zhang J., Tang Z., Yao Z., Liu Y., Liu X. (2019). Calcium-Overload-Mediated Tumor Therapy by Calcium Peroxide Nanoparticles. Chem.

[29] Kamba A.S., Ismail M., Ibrahim T.A.T., Zakaria Z.A.B. (2013). A pH-Sensitive, Biobased Calcium Carbonate Aragonite Nanocrystal as a Novel Anticancer Delivery System. BioMed Res. Int..

[30] Islam K.N., Zuki A.B.Z., Ali M.E., Bin Hussein M.Z., Noordin M.M., Loqman M.Y., Wahid H., Hakim M.A., Hamid S.B.A. (2012). Facile Synthesis of Calcium Carbonate Nanoparticles from Cockle Shells. J. Nanomater..

[31] Ueno Y., Futagawa H., Takagi Y., Ueno A., Mizushima Y. (2005). Drug-incorporating calcium carbonate nanoparticles for a new delivery system. J Control. Release.

[32] Parakhonskiy B., Zyuzin M.V., Yashchenok A., Carregal-Romero S., Rejman J., Möhwald H., Parak W.J., Skirtach A.G. (2015). The influence of the size and aspect ratio of anisotropic, porous CaCO_3_ particles on their uptake by cells. J. Nanobiotechnol..

[33] Feng Q., Zhang W., Yang X., Li Y., Hao Y., Zhang H., Hou L., Zhang Z. (2018). pH/Ultrasound Dual-Responsive Gas Generator for Ultrasound Imaging-Guided Therapeutic Inertial Cavitation and Sonodynamic Therapy. Adv. Health Mater..

[34] Ruan H., Hu Q., Wen D., Chen Q., Chen G., Lu Y., Wang J., Cheng H., Lu W., Gu Z. (2019). A Dual-Bioresponsive Drug-Delivery Depot for Combination of Epigenetic Modulation and Immune Checkpoint Blockade. Adv. Mater..

[35] Yao M., Han W., Feng L., Wei Z., Liu Y., Zhang H., Zhang S. (2022). pH-programmed responsive nanoplatform for synergistic cancer therapy based on single atom catalysts. Eur. J. Med. Chem..

[36] Huo W., Yang X., Wang B., Cao L., Fang Z., Li Z., Liu H., Liang X.-J., Zhang J., Jin Y. (2022). Biomineralized hydrogel DC vaccine for cancer immunotherapy: A boosting strategy via improving immunogenicity and reversing immune-inhibitory microenvironment. Biomaterials.

[37] Xie W., Ye J., Guo Z., Lu J., Xu W., Gao X., Huang H., Hu R., Mao L., Wei Y. (2022). TME-responded Full-biodegradable nanocatalyst for mitochondrial calcium Overload-induced hydroxyl radical bursting cancer treatment. Chem. Eng. J..

[38] Zhang Y., Cai L., Li D., Lao Y.-H., Liu D., Li M., Ding J., Chen X. (2018). Tumor microenvironment-responsive hyaluronate-calcium carbonate hybrid nanoparticle enables effective chemotherapy for primary and advanced osteosarcomas. Nano Res..

[39] Kim H.J., Min K.H., Lee H.J., Hwang Y.-S., Lee S.C. (2019). Fenton-like reaction performing mineralized nanocarriers as oxidative stress amplifying anticancer agents. J. Ind. Eng. Chem..

[40] Yang S., Zhang Y., Lu S., Yang L., Yu S., Yang H. (2021). CaCO_3_-Encapsulated Au Nanoparticles Modulate Macrophages toward M1-like Phenotype. ACS Appl. Bio Mater..

[41] An J., Zhang K., Wang B., Wu S., Wang Y., Zhang H., Zhang Z., Liu J., Shi J. (2020). Nanoenabled Disruption of Multiple Barriers in Antigen Cross-Presentation of Dendritic Cells via Calcium Interference for Enhanced Chemo-Immunotherapy. ACS Nano.

[42] Li K., Li D., Zhao L., Chang Y., Zhang Y., Cui Y., Zhang Z. (2020). Calcium-mineralized polypeptide nanoparticle for intracellular drug delivery in osteosarcoma chemotherapy. Bioact. Mater..

[43] Guan Q., Zhou L., Lv F., Li W., Li Y., Dong Y. (2020). A Glycosylated Covalent Organic Framework Equipped with BODIPY and CaCO_3_ for Synergistic Tumor Therapy. Angew. Chem. Int. Ed..

[44] Zhao Q., Gong Z., Li Z., Wang J., Zhang J., Zhao Z., Zhang P., Zheng S., Miron R.J., Yuan Q. (2021). Target Reprogramming Lysosomes of CD8+ T Cells by a Mineralized Metal-Organic Framework for Cancer Immunotherapy. Adv. Mater..

[45] Wang P., Xue J., Wu S., Pei Y., Xu L., Wang Y. (2021). Cell-Friendly Isolation and pH-Sensitive Controllable Release of Circulating Tumor Cells by Fe_3_O_4_@CaCO_3_ Nanoplatform. Adv. Mater. Interfaces.

[46] Wang S., Ni D., Yue H., Luo N., Xi X., Wang Y., Shi M., Wei W., Ma G. (2018). Exploration of Antigen Induced CaCO_3_ Nanoparticles for Therapeutic Vaccine. Small.

[47] Vidallon M.L.P., Douek A.M., Quek A., McLiesh H., Kaslin J., Tabor R.F., Bishop A.I., Teo B.M. (2020). Gas-Generating, pH-Responsive Calcium Carbonate Hybrid Particles with Biomimetic Coating for Contrast-Enhanced Ultrasound Imaging. Part. Part. Syst. Charact..

[48] Ju Y., Zhao Y., Guan Q., Yang S., Wang W., Yan B., Meng Y., Li S., Tang P., Mao L. (2022). Amorphous Calcium Carbonate Cluster Nanospheres in Water-Deficient Organic Solvents. Angew. Chem. Int. Ed..

[49] Zhao Y., Luo Z., Li M., Qu Q., Ma X., Yu S.H., Zhao Y. (2015). A Preloaded Amorphous Calcium Carbonate/Doxorubicin@Silica Nanoreactor for pH-Responsive Delivery of an Anticancer Drug. Angew. Chem. Int. Ed..

[50] Wang C., Chen S., Wang Y., Liu X., Hu F., Sun J., Yuan H. (2018). Lipase-Triggered Water-Responsive “Pandora’s Box” for Cancer Therapy: Toward Induced Neighboring Effect and Enhanced Drug Penetration. Adv. Mater..

[51] Zhang M.K., Li C.X., Wang S.B., Liu T., Song X.L., Yang X.Q., Feng J., Zhang X.-Z. (2018). Tumor Starvation Induced Spatiotemporal Control over Chemotherapy for Synergistic Therapy. Small.

[52] Huang X., Wu J., He M., Hou X., Wang Y., Cai X., Xin H., Gao F., Chen Y. (2019). Combined Cancer Chemo-Photodynamic and Photothermal Therapy Based on ICG/PDA/TPZ-Loaded Nanoparticles. Mol. Pharm..

[53] Liu X., Wang C., Ma H., Yu F., Hu F., Yuan H. (2019). Water-Responsive Hybrid Nanoparticles Codelivering ICG and DOX Effec-tively Treat Breast Cancer via Hyperthermia-aided DOX Functionality and Drug Penetration. Adv. Healthc. Mater..

[54] Wang C., Chen S., Yu F., Lv J., Zhao R., Hu F., Yuan H. (2021). Dual-Channel Theranostic System for Quantitative Self-Indication and Low-Temperature Synergistic Therapy of Cancer. Small.

[55] Wan X., Zhong H., Pan W., Li Y., Chen Y., Li N., Tang B. (2019). Programmed Release of Dihydroartemisinin for Synergistic Cancer Therapy Using a CaCO_3_ Mineralized Metal–Organic Framework. Angew. Chem. Int. Ed..

[56] Chang M., Hou Z., Jin D., Zhou J., Wang M., Wang M., Shu M., Ding B., Li C., Lin J. (2020). Colorectal Tumor Microenvironment-Activated Bio-Decomposable and Metabolizable Cu_2_O@CaCO_3_ Nanocomposites for Synergistic Oncotherapy. Adv. Mater..

[57] Dong Z., Feng L., Hao Y., Li Q., Chen M., Yang Z., Zhao H., Liu Z. (2020). Synthesis of CaCO_3_-Based Nanomedicine for Enhanced Sonodynamic Therapy via Amplification of Tumor Oxidative Stress. Chem.

[58] Dong Z., Hao Y., Li Q., Yang Z., Zhu Y., Liu Z., Feng L. (2020). Metal-polyphenol-network coated CaCO_3_ as pH-responsive nanocarriers to enable effective intratumoral penetration and reversal of multidrug resistance for augmented cancer treatments. Nano Res..

[59] Li Y., Gong S., Pan W., Chen Y., Liu B., Li N., Tang B. (2020). A tumor acidity activatable and Ca2+-assisted immuno-nanoagent enhances breast cancer therapy and suppresses cancer recurrence. Chem. Sci..

[60] Wang M., Zhou B., Wang L., Zhou F., Smith N., Saunders D., Towner R.A., Song J., Qu J., Chen W.R. (2020). Biodegradable pH-responsive amorphous calcium carbonate nanoparticles as immunoadjuvants for multimodal imaging and enhanced photoimmunotherapy. J. Mater. Chem. B.

[61] Li Y., Zhou S., Song H., Yu T., Zheng X., Chu Q., Wang Y. (2021). CaCO_3_ nanoparticles incorporated with KAE to enable amplified calcium overload cancer therapy. Biomaterials.

[62] Xue C., Li M., Sutrisno L., Yan B., Zhao Y., Hu Y., Cai K., Zhao Y., Yu S., Luo Z. (2021). Bioresorbable Scaffolds with Biocatalytic Chemotherapy and In Situ Microenvironment Modulation for Postoperative Tissue Repair. Adv. Funct. Mater..

[63] Zheng C., Song Q., Zhao H., Kong Y., Sun L., Liu X., Feng Q., Wang L. (2021). A nanoplatform to boost multi-phases of cancer-immunity-cycle for enhancing immunotherapy. J. Control. Release.

[64] Zheng P., Ding B., Jiang Z., Xu W., Li G., Ding J., Chen X. (2021). Ultrasound-Augmented Mitochondrial Calcium Ion Overload by Calcium Nanomodulator to Induce Immunogenic Cell Death. Nano Lett..

[65] Zheng P., Ding B., Shi R., Jiang Z., Xu W., Li G., Ding J., Chen X. (2021). A Multichannel Ca2+ Nanomodulator for Multilevel Mito-chondrial Destruction-Mediated Cancer Therapy. Adv. Mater..

[66] Feng Y., Qin R., Xu L., Ma X., Ding D., Li S., Chen L., Liu Y., Sun W., Chen H. (2022). Ion drugs for precise orthotopic tumor management by in situ the generation of toxic ion and drug pools. Theranostics.

[67] Gao H., Wang Z., Tan M., Liu W., Zhang L., Huang J., Cao Y., Li P., Wang Z., Wen J. (2022). pH-Responsive Nano-particles for Enhanced Antitumor Activity by High-Intensity Focused Ultrasound Therapy Combined with Sonodynamic Therapy. Int. J. Nanomed..

[68] Ma X., Wang C., Dong Z., Hu C., Feng L. (2022). Lipid-coated CaCO_3_-PDA nanoparticles as a versatile nanocarrier to enable pH-responsive dual modal imaging-guided combination cancer therapy. J. Mater. Chem. B.

[69] Shen J., Liao X., Wu W., Feng T., Karges J., Lin M., Luo H., Chen Y., Chao H. (2022). A pH-responsive iridium(iii) two-photon photosensitizer loaded CaCO_3_ nanoplatform for combined Ca2+ overload and photodynamic therapy. Inorg. Chem. Front..

[70] Wu J., Cai X., Williams G.R., Meng Z., Zou W., Yao L., Hu B., Chen Y., Zheng Y. (2022). 2D antimonene-integrated composite nanomedicine for augmented low-temperature photonic tumor hyperthermia by reversing cell thermoresistance. Bioact. Mater..

[71] Xu M., Zhang J., Mu Y., Foda M.F., Han H. (2022). Activation of TRPV1 by capsaicin-loaded CaCO_3_ nanoparticle for tumor-specific therapy. Biomaterials.

[72] Zhou Y., Jing S., Liu S., Shen X., Cai L., Zhu C., Zhao Y., Pang M. (2022). Double-activation of mitochondrial permeability transition pore opening via calcium overload and reactive oxygen species for cancer therapy. J. Nanobiotechnol..

[73] Zheng P., Ding B., Zhu G., Li C., Lin J. (2022). Biodegradable Ca2+ Nanomodulators Activate Pyroptosis through Mitochondrial Ca2+ Overload for Cancer Immunotherapy. Angew. Chem. Int. Ed..

[74] Deng G., Wu Y., Song Z., Li S., Du M., Deng J., Xu Q., Deng L., Bahlol H.S., Han H. (2022). Tea Polyphenol Liposomes Overcome Gastric Mucus to Treat Helicobacter Pylori Infection and Enhance the Intestinal Microenvironment. ACS Appl. Mater. Interfaces.

[75] Stawski T.M., Roncal-Herrero T., Fernandez-Martinez A., Matamoros-Veloza A., Kröger R., Benning L.G. (2018). On demand” triggered crystallization of CaCO_3_ from solute precursor species stabilized by the water-in-oil microemulsion. Phys. Chem. Chem. Phys..

[76] Shen Y., Xie A., Chen Z., Xu W., Yao H., Li S., Huang L., Wu Z., Kong X. (2007). Controlled synthesis of calcium carbonate nanocrystals with multi-morphologies in different bicontinuous microemulsions. Mater. Sci. Eng. A.

[77] Jang H.J., Jeong E.J., Lee K.Y. (2018). Carbon Dioxide-Generating PLG Nanoparticles for Controlled Anti-Cancer Drug Delivery. Pharm. Res..

[78] Zhao P., Li M., Wang Y., Chen Y., He C., Zhang X., Yang T., Lu Y., You J., Lee R.J. (2018). Enhancing anti-tumor efficiency in hepatocellular carcinoma through the autophagy inhibition by miR-375/sorafenib in lipid-coated calcium carbonate nanoparticles. Acta Biomater..

[79] Chen Y., Du Q., Guo Q., Huang J., Liu L., Shen X., Peng J. (2019). A W/O emulsion mediated film dispersion method for curcumin encapsulated pH-sensitive liposomes in the colon tumor treatment. Drug Dev. Ind. Pharm..

[80] Khan M.W., Zhao P., Khan A., Raza F., Raza S.M., Sarfraz M., Chen Y., Li M., Yang T., Ma X. (2019). Synergism of cisplatin-oleanolic acid co-loaded calcium carbonate nanoparticles on hepatocellular carcinoma cells for enhanced apoptosis and reduced hepatotoxicity. Int. J. Nanomed..

[81] Wei K., Zhang J., Li X., Shi P., Fu P. (2019). High density lipoprotein coated calcium carbonate nanoparticle for chemotherapy of breast cancer. J. Biomater. Appl..

[82] Li H., Zhang X., Lin X., Zhuang S., Wu Y., Liu Z. (2020). CaCO_3_ nanoparticles pH-sensitively induce blood coagulation as a potential strategy for starving tumor therapy. J. Mater. Chem. B.

[83] Zhu Y., Yang Z., Dong Z., Gong Y., Hao Y., Tian L., Yang X., Liu Z., Feng L. (2020). CaCO_3_-Assisted Preparation of pH-Responsive Immune-Modulating Nanoparticles for Augmented Chemo-Immunotherapy. Nano-Micro Lett..

[84] Khan M.W., Zou C., Hassan S., Din F.U., Razak M.Y.A., Nawaz A., Alam Z., Wahab A., Bangash S.A. (2022). Cisplatin and oleanolic acid Co-loaded pH-sensitive CaCO_3_ nanoparticles for synergistic chemotherapy. RSC Adv..

[85] Liu Y., Ma X., Zhu Y., Lv X., Wang P., Feng L. (2022). pH-responsive nanomedicine co-encapsulated with Erlotinib and chlorin e6 can enable effective treatment of triple negative breast cancer via reprogramming tumor vasculature. Chem. Eng. J..

[86] Hu Z., Deng Y., Sun Q. (2004). Synthesis of precipitated calcium carbonate nanoparticles using a two-membrane system. Colloid J..

[87] Hamidu A., Mokrish A., Mansor R., Razak I.S.A., Danmaigoro A., Jaji A.Z., Bakar Z.A. (2019). Modified methods of nanoparticles synthesis in pH-sensitive nano-carriers production for doxorubicin delivery on MCF-7 breast cancer cell line. Int. J. Nanomed..

[88] Jangili P., Kong N., Kim J.H., Zhou J., Liu H., Zhang X., Tao W., Kim J.S. (2022). DNA-Damage-Response-Targeting Mitochon-dria-Activated Multifunctional Prodrug Strategy for Self-Defensive Tumor Therapy. Angew. Chem. Int. Ed..

[89] Yang Z., Gao D., Guo X., Jin L., Zheng J., Wang Y., Chen S., Zheng X., Zeng L., Guo M. (2020). Fighting Immune Cold and Reprogramming Immunosuppressive Tumor Microenvironment with Red Blood Cell Membrane-Camouflaged Nanobullets. ACS Nano.

[90] Xue C.-C., Li M.-H., Zhao Y., Zhou J., Hu Y., Cai K.-Y., Zhao Y., Yu S.-H., Luo Z. (2020). Tumor microenvironment-activatable Fe-doxorubicin preloaded amorphous CaCO_3_ nanoformulation triggers ferroptosis in target tumor cells. Sci. Adv..

[91] Wang C., Dong Z., Hao Y., Zhu Y., Ni J., Li Q., Liu B., Han Y., Yang Z., Wan J. (2022). Coordination Polymer-Coated CaCO_3_ Reinforces Radiotherapy by Reprogramming the Immunosuppressive Metabolic Microenvironment. Adv. Mater..

[92] Chen Q., Wang C., Zhang X., Chen G., Hu Q., Li H., Wang J., Wen D., Zhang Y., Lu Y. (2019). In situ sprayed bioresponsive immunotherapeutic gel for post-surgical cancer treatment. Nat. Nanotechnol..

[93] Guo D., Dai X., Liu K., Liu Y., Wu J., Wang K., Jiang S., Sun F., Wang L., Guo B. (2023). A Self-Reinforcing Nanoplatform for Highly Effective Synergistic Targeted Combinatary Calcium-Overload and Photodynamic Therapy of Cancer. Adv. Healthc. Mater..

[94] Orrenius S., Zhivotovsky B., Nicotera P. (2003). Regulation of cell death: The calcium–apoptosis link. Nat. Rev. Mol. Cell Biol..

[95] Chen Q., Zhou J., Chen Z., Luo Q., Xu J., Song G. (2019). Tumor-Specific Expansion of Oxidative Stress by Glutathione Depletion and Use of a Fenton Nanoagent for Enhanced Chemodynamic Therapy. ACS Appl. Mater. Interfaces.

[96] Ermak G., Davies K.J. (2002). Calcium and oxidative stress: From cell signaling to cell death. Mol. Immunol..

[97] Zhu J., Jiao A., Li Q., Lv X., Wang X., Song X., Li B., Zhang Y., Dong X. (2022). Mitochondrial Ca^2+^-overloading by oxygen/glutathione depletion-boosted photodynamic therapy based on a CaCO_3_ nanoplatform for tumor synergistic therapy. Acta Biomater..

[98] He W., Xing X., Wang X., Wu D., Wu W., Guo J., Mitragotri S. (2020). Nanocarrier-Mediated Cytosolic Delivery of Biopharmaceuticals. Adv. Funct. Mater..

[99] Vermeulen L.M., De Smedt S.C., Remaut K., Braeckmans K. (2018). The proton sponge hypothesis: Fable or fact?. Eur. J. Pharm. Biopharm..

[100] Mindell J.A. (2012). Lysosomal Acidification Mechanisms. Annu. Rev. Physiol..

[101] Hao Y., Chen M., Wu Y., Dong Z., Zhu Y., Wang C., Li Q., Yang Z., Liu Z., Feng L. (2023). CaCO_3_ based proton nanosponge to potentiate immune checkpoint blockade therapy by synergistically reversing tumor immunosuppression. Chem. Eng. J..

[102] Zhou X., Huang X., Wang B., Tan L., Zhang Y., Jiao Y. (2021). Light/gas cascade-propelled Janus micromotors that actively overcome sequential and multi-staged biological barriers for precise drug delivery. Chem. Eng. J..

[103] Bai S., Lan Y., Fu S., Cheng H., Lu Z., Liu G. (2022). Connecting Calcium-Based Nanomaterials and Cancer: From Diagnosis to Therapy. Nano-Micro Lett..

[104] An J., Liu M., Zhao L., Lu W., Wu S., Zhang K., Liu J., Zhang Z., Shi J. (2022). Boosting Tumor Immunotherapy by Bioactive Nanoparticles via Ca^2+^ Interference Mediated TME Reprogramming and Specific PD-L1 Depletion. Adv. Funct. Mater..

[105] Kang H., Zhang K., Wong D.S.H., Han F., Li B., Bian L. (2018). Near-infrared light-controlled regulation of intracellular calcium to modulate macrophage polarization. Biomaterials.

[106] Galluzzi L., Buqué A., Kepp O., Zitvogel L., Kroemer G. (2017). Immunogenic cell death in cancer and infectious disease. Nat. Rev. Immunol..

[107] Dai Z., Tang J., Gu Z., Wang Y., Yang Y., Yang Y., Yu C. (2020). Eliciting Immunogenic Cell Death via a Unitized Nanoinducer. Nano Lett..

[108] Shi Y., Lin G., Zheng H., Mu D., Chen H., Lu Z., He P., Zhang Y., Liu C., Lin Z. (2021). Biomimetic nanoparticles blocking autophagy for enhanced chemotherapy and metastasis inhibition via reversing focal adhesion disassembly. J. Nanobiotechnol..

[109] Shi Y., Wang J., Liu J., Lin G., Xie F., Pang X., Pei Y., Cheng Y., Zhang Y., Lin Z. (2020). Oxidative stress-driven DR5 upregulation restores TRAIL/Apo2L sensitivity induced by iron oxide nanoparticles in colorectal cancer. Biomaterials.

[110] Guan Y.H., Wang N., Deng Z.W., Chen X.G., Liu Y. (2022). Exploiting autophagy-regulative nanomaterials for activation of den-dritic cells enables reinforced cancer immunotherapy. Biomaterials.

[111] Crawford S.E., Estes M.K. (2013). Viroporin-mediated calcium-activated autophagy. Autophagy.

[112] Casanova-Acebes M., Dalla E., Leader A.M., LeBerichel J., Nikolic J., Morales B.M., Brown M., Chang C., Troncoso L., Chen S.T. (2021). Tissue-resident macrophages provide a pro-tumorigenic niche to early NSCLC cells. Nature.

[113] Mosser D.M., Hamidzadeh K., Goncalves R. (2021). Macrophages and the maintenance of homeostasis. Cell. Mol. Immunol..

[114] Qiu Y., Chen T., Hu R., Zhu R., Li C., Ruan Y., Xie X., Li Y. (2021). Next frontier in tumor immunotherapy: Macrophage-mediated immune evasion. Biomark. Res..

[115] Mantovani A., Marchesi F., Malesci A., Laghi L., Allavena P. (2017). Tumour-associated macrophages as treatment targets in oncology. Nat. Rev. Clin. Oncol..

[116] Chen D., Xie J., Fiskesund R., Dong W., Liang X., Lv J., Jin X., Liu J., Mo S., Zhang T. (2018). Chloroquine modulates antitumor immune response by resetting tumor-associated macrophages toward M1 phenotype. Nat. Commun..

[117] Zhou P., Xia D., Ni Z., Ou T., Wang Y., Zhang H., Mao L., Lin K., Xu S., Liu J. (2021). Calcium silicate bioactive ceramics induce osteogenesis through oncostatin M. Bioact. Mater..

[118] Park D.J., Min K.H., Lee H.J., Kim K., Kwon I.C., Jeong S.Y., Lee S.C. (2016). Photosensitizer-loaded bubble-generating mineralized nanoparticles for ultrasound imaging and photodynamic therapy. J. Mater. Chem. B.

[119] Chen S., Xu X.L., Zhou B., Tian J., Luo B.M., Zhang L.M. (2019). Acidic pH-Activated Gas-Generating Nanoparticles with Pullulan Decorating for Hepatoma-Targeted Ultrasound Imaging. ACS Appl. Mater. Interfaces.

[120] Huang H., Zhang W., Liu Z., Guo H., Zhang P. (2020). Smart responsive-calcium carbonate nanoparticles for dual-model cancer imaging and treatment. Ultrasonics.

[121] Yi Z., Luo Z., Barth N.D., Meng X., Liu H., Bu W., All A., Vendrell M., Liu X. (2019). In Vivo Tumor Visualization through MRI Off-On Switching of NaGdF_4_-CaCO_3_ Nanoconjugates. Adv. Mater..

[122] Zhang C., Li S., Yu A., Wang Y. (2019). Nano CaCO_3_ “Lysosomal Bombs” Enhance Chemotherapy Drug Efficacy via Rebalancing Tumor Intracellular pH. ACS Biomater. Sci. Eng..

[123] Xue P., Hou M., Sun L., Li Q., Zhang L., Xu Z., Kang Y. (2018). Calcium-carbonate packaging magnetic polydopamine nanoparticles loaded with indocyanine green for near-infrared induced photothermal/photodynamic therapy. Acta Biomater..

[124] Liu B., Cao W., Cheng J., Fan S., Pan S., Wang L., Niu J., Pan Y., Liu Y., Sun X. (2019). Human natural killer cells for targeting delivery of gold nanostars and bimodal imaging directed photothermal/photodynamic therapy and immunotherapy. Cancer Biol. Med..

[125] Liu W., Li Z., Qiu Y., Li J., Yang J., Li J. (2021). Biomineralization of Aggregation-Induced Emission-Active Photosensitizers for pH-Mediated Tumor Imaging and Photodynamic Therapy. ACS Appl. Bio Mater..

[126] Yang S., Xue Y., Zhang Y., Lu S., Wu J., Yang L., Yang H., Yu S. (2021). Mitochondrial Targeting Strategy for Enhanced Photothermal Cancer Therapy. ChemNanoMat.

[127] Tan H., Liu Y., Hou N., Cui S., Liu B., Fan S., Yu G., Han C., Zheng D., Li W. (2022). Tumor microenvironment pH-responsive pentagonal gold prism-based nanoplatform for multimodal imaging and combined therapy of castration-resistant prostate cancer. Acta Biomater..

[128] Xie M., Zhu Y., Xu S., Xu G., Xiong R., Sun X., Liu C. (2020). A nanoplatform with tumor-targeted aggregation and drug-specific release characteristics for photodynamic/photothermal combined antitumor therapy under near-infrared laser irradiation. Nanoscale.

[129] Liu Y., Yu B., Dai X., Zhao N., Xu F.-J. (2021). Biomineralized calcium carbonate nanohybrids for mild photothermal heating-enhanced gene therapy. Biomaterials.

[130] Ding X.-L., Liu M.-D., Cheng Q., Guo W.-H., Niu M.-T., Huang Q.-X., Zeng X., Zhang X.-Z. (2022). Multifunctional liquid metal-based nanoparticles with glycolysis and mitochondrial metabolism inhibition for tumor photothermal therapy. Biomaterials.

[131] Han Y., Dong Z., Wang C., Li Q., Hao Y., Yang Z., Zhu W., Zhang Y., Liu Z., Feng L. (2022). Ferrous ions doped calcium carbonate nanoparticles potentiate chemotherapy by inducing ferroptosis. J. Control. Release.

[132] Xu Y., Wang C., Shen F., Dong Z., Hao Y., Chen Y., Liu Z., Feng L. (2022). Lipid-Coated CaCO_3_ Nanoparticles as a Versatile pH-Responsive Drug Delivery Platform to Enable Combined Chemotherapy of Breast Cancer. ACS Appl. Bio Mater..

[133] Yang C., Gao M., Zhao H., Liu Y., Gao N., Jing J., Zhang X. (2021). A dual-functional biomimetic-mineralized nanoplatform for glucose detection and therapy with cancer cells in vitro. J. Mater. Chem. B.

[134] Wang W., Zhao Y., Yan B.-B., Dong L., Lu Y., Yu S.-H. (2018). Calcium carbonate-doxorubicin@silica-indocyanine green nanospheres with photo-triggered drug delivery enhance cell killing in drug-resistant breast cancer cells. Nano Res..

[135] Wang C., Yu F., Liu X., Chen S., Wu R., Zhao R., Hu F., Yuan H. (2021). Cancer-Specific Therapy by Artificial Modulation of Intracellular Calcium Concentration. Adv. Healthc. Mater..

[136] Sheng Y., Gao J., Yin Z.-Z., Kang J., Kong Y. (2021). Dual-drug delivery system based on the hydrogels of alginate and sodium carboxymethyl cellulose for colorectal cancer treatment. Carbohydr. Polym..

[137] Wu Y., Gu W., Tang J., Xu Z.P. (2017). Devising new lipid-coated calcium phosphate/carbonate hybrid nanoparticles for controlled release in endosomes for efficient gene delivery. J. Mater. Chem. B.

[138] Wang Y., Zhao K., Xie L., Li K., Zhang W., Xi Z., Wang X., Xia M., Xu L. (2022). Construction of calcium carbonate-liposome dual-film coated mesoporous silica as a delayed drug release system for antitumor therapy. Colloids Surf. B Biointerfaces.

